# Designing Zinc Deposition Substrate with Fully Preferred Orientation to Elude the Interfacial Inhomogeneous Dendrite Growth

**DOI:** 10.34133/2022/9841343

**Published:** 2022-08-18

**Authors:** Chunlin Xie, Zefang Yang, Qi Zhang, Huimin Ji, Yihu Li, Tingqing Wu, Wenbin Li, Pengfei Wu, Haiyan Wang

**Affiliations:** Hunan Provincial Key Laboratory of Chemical Power Sources, College of Chemistry and Chemical Engineering, Central South University, Changsha 410083, China

## Abstract

The development of zinc-ion batteries with high energy density remains a great challenge due to the uncontrollable dendrite growth on their zinc metal anodes. Film anodes plated on the substrate have attracted increasing attention to alleviate these dendrite issues. Herein, we first point out that both the random crystal orientation and the low metal affinity of the substrate are important factors of zinc dendrite formation. Accordingly, the (1 0 1) fully preferred tin interface layer with high zinc affinity was fabricated by chemical tin plating on (1 0 0) oriented copper. This tin decorated copper substrate can realize high reversible zinc plating/stripping behavior, and full cell using this zinc plated substrate can be operated for more than 1000 cycles with high capacity retention (85.3%) and low electrochemical impedance. The proposed strategy can be also applied to lithium metal batteries, which demonstrates that the substrate orientation regulation and metal affinity design are the promising approaches to achieve dendrite-free metal anode and overcome the challenges of highly reactive metal anodes.

## 1. Introduction

The high-energy metals (e.g., lithium, sodium, and zinc) are considered to be the most promising anode materials to realize the next generation of high-energy-density batteries [1, 2]. Among them, zinc metal can exhibit high theoretical specific capacity (820 mAh·g^−1^), low redox potential (−0.76 V versus standard hydrogen electrode), abundant natural resources, and low manufacturing cost, which endows aqueous zinc-ion batteries with great potential to be applied in large-scale energy storage systems [3, 4]. However, the practicality of zinc metal batteries is hindered by the formation of rough and nonuniform electrodeposits, which results in the continuous loss of active material through multiple mechanisms [5–7]. This phenomenon is also dangerous because when uneven or dendritic metal deposits grow in the inner space of the battery and bridge the cathode and anode, the induced short circuit can trigger the battery to burst [8, 9].

Numerous efforts have been devoted to alleviating these zinc-related issues, including modulation of zinc anodes (e.g., zinc alloy and protective layer), optimization of electrolyte formulations, and design of current collectors [10–14]. Designing current collectors is considered a feasible approach to improve zinc plating/stripping reversibility in comparison to the common hostless zinc foil anodes with low zinc utilization [15, 16]. Commercial copper substrates were intensively investigated for zinc deposition because they are the immediately available primary material for industrial manufacture [10, 17, 18]. However, the zinc anodes always fail quickly when using pristine copper current collectors, and their mechanism towards zinc dendrite growth is still incompletely understood [19]. Most researchers focus on constructing a thin layer of foreign substances with high zinc affinity (such as tin and silver) to reduce nucleation barriers and promote uniform zinc growth during cycling but overlook its intrinsic factors affecting metal affinity [20–22]. We have proved in our previous work that zinc interacts differently with different crystal facets of a foreign substance [23]. There is an obvious difference in zinc adsorption on the randomly oriented substrate with high zinc affinity, which enables zinc to be deposited preferentially on the facet with stronger zinc affinity on the surface with random orientation, resulting in uneven zinc growth. Considering the facet-selective lithium deposition on a randomly oriented copper substrate, the orientation heterogeneity of the interface may also be one of the main reasons for the inhomogeneous zinc deposition [24, 25]. Besides, uniformly oriented graphene coating layer with low lattice mismatch to zinc can induce highly reversible zinc electrodeposition, but its zinc affinity is insufficient [20, 26]. Accordingly, the construction of a substrate surface with both high zinc affinity and uniform crystal orientation may be a more effective strategy to improve the cycling stability of electroplated zinc anodes in practical batteries.

In this work, we reveal the selective zinc deposition behavior on polycrystalline copper foil with random crystal orientation and further prove by theoretical simulations that the orientation difference between grains on the surface is an important factor in inducing uneven electric fields. A strategy to construct a uniformly oriented substrate surface is proposed to induce homogeneous zinc deposition based on theoretical simulation prediction. A copper-supported (1 0 1) fully preferred tin (F-Sn@Cu) substrate is first fabricated by chemical tin plating technology, in which the (1 0 0) fully preferred copper (F-Cu) for tin plating is obtained by low-temperature annealing of commercial copper foils. The as-prepared F-Sn@Cu substrate with high zinc affinity exhibits highly reversible zinc plating/stripping behavior, compact deposition appearance, and enhanced hydrogen evolution resistance. It is also demonstrated that this substrate with fully preferred crystal orientation can be easily extended to lithium metal anodes. The strategy of orientation regulation and metal affinity design of interfaces provides research insights for designing stable current collectors to resist the dendrite growth on active metal anodes.

## 2. Results and Discussion

### 2.1. Influence of the Substrate Crystal Orientations on Zinc Deposition

**Figure 1:**
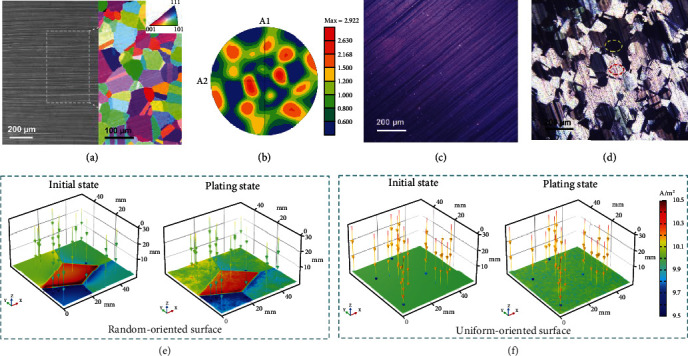
The selective zinc deposition on the specific copper foil with a large grain size. (a) Scanning electron microscope (SEM), the corresponding inverse pole figure (IPF) map, and (b) (0 0 1) pole figure of the specific copper foil. Optical patterns of the specific copper foil (c) before and (d) after zinc deposition at 5 mA cm^−2^ for 20 s. The regions cycled by red and yellow refer to high-activity facet and inert facet, respectively. Finite element simulations of electric field on the surface with (e) random and (f) uniform orientation.

The zinc deposition behavior influenced by the substrate crystal orientation was investigated on a specific commercial copper foil with large grain size and random orientation (Figures 1(a) and 1(b) and S1, S2). Interestingly, there is a significant difference in the amount of zinc deposition on different grains of the substrate surface after zinc deposition at 5 mA cm^−2^ for 20 s (Figures 1(c) and 1(d)). Most of the zinc tends to be preferentially deposited on the high-activity facets, as evidenced by the metallic luster on the smooth plating layer. And there exist several inert facets without electrochemical deposition reaction, which can be explained by the difference in activity on different crystalline facets of the randomly oriented copper substrate surface. Such facet dependency of zinc deposition was further proved by energy dispersive spectroscopy and electron back-scattered diffraction (EBSD) analysis (Figure S3). It has been demonstrated in some previous reports that different crystal facets exhibit different exchange current densities (ECD), which can reflect the difficulty of the electrode reaction [27, 28]. Accordingly, the effect of the randomly oriented surfaces with different ECD on the zinc deposition behavior is simulated by the finite element method (Table S1). The surfaces with different ECD show an uneven electric field in the initial state, which leads to the selective zinc deposition on the high electric field region and further aggravates the uneven electric field distribution during the continuous zinc plating process (Figure 1(e)). The selective zinc deposition rather than the overall zinc deposition on the interface evolves into uncontrollable dendrite growth and induces rapid cell failure (Figure S4 and S5) [29, 30]. These experimental results and theoretical simulations confirm that selective zinc deposition is affected by the crystal orientation of substrate surfaces, which may be an important factor in dendrite formation. Therefore, it can be inferred that if the crystal orientation of the substrate surface is uniform, a complete and homogeneous zinc deposition can be achieved owing to the uniform electric field distribution on the uniformly oriented surface without ECD difference (Figure 1(f)).

### 2.2. Fabrication and Characterization of F-Cu and F-Sn@Cu

**Figure 2:**
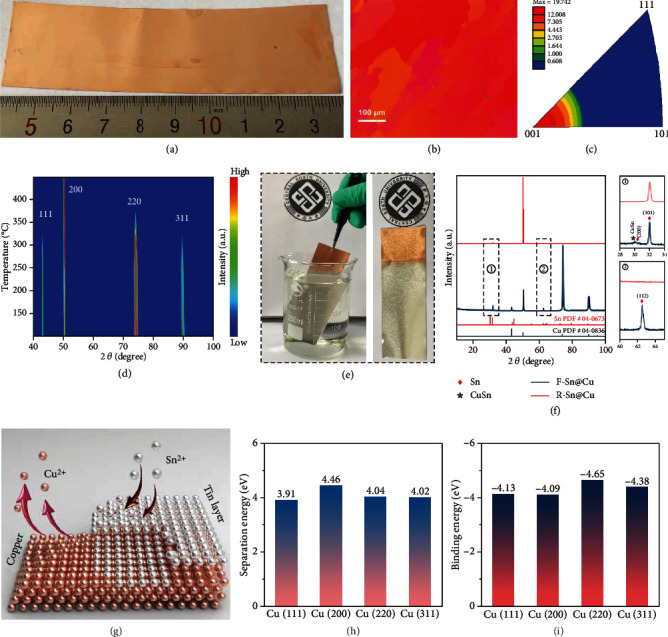
Preparation and structure characterization of F-Cu and F-Sn@Cu substrates. (a) Digital photograph, (b) IPF map image, and (c) the inverse pole figure in (0 0 1) direction of the common commercial copper foil after annealing. (d) In situ XRD patterns of the common commercial copper foil at elevated temperature. (e) Schematic illustration of chemical tin plating process. (f) XRD patterns of F-Sn@Cu and R-Sn@Cu substrates. (g) Schematic illustration of the chemical tin plating on the copper foil. (h) Separation energy of copper atom from different copper facets and (i) binding energy of tin on the different copper facets.

A low-temperature annealing method was applied to convert polycrystalline grains with random orientation into a uniform orientation in a common commercial copper foil. As seen in the X-ray diffraction (XRD) pattern (Figure S6), the (2 0 0) fully preferred copper is observed after the copper foil was annealed at 450°C, which implies that the crystal orientation of the copper foil surface was reconstructed after low-temperature annealing [31]. There is no obvious change in the annealed copper foil at both the microscopic and macroscopic levels (Figure 2(a) and Figure S7, S8). The crystal orientation shift toward (1 0 0) orientation in the annealed copper foils is further confirmed by the EBSD measurement. The annealed copper foil shows the relatively uniform color close to red in the IPF map (Figure 2(b)) and the obvious (0 0 1) orientation characteristic in the inverse pole figure (Figure 2(c)) in comparison to the pristine copper foil with a colorful IPF map and random directions in the inverse pole figure (Figure S9). It is well known that the (1 1 1) facet of copper is thermodynamically stable because the close-packed copper (1 1 1) facet has the lowest surface energy among all crystal facets in the face-centered cubic structure [32, 33]. However, the stable copper structure may be different if the driving force for grain growth is not mainly surface energy but other energy (such as strain energy caused by thermal stress) [34]. In situ XRD clearly shows the crystal orientation shift process of copper foil during the low-temperature annealing (Figure 2(d)). The intensity of (2 0 0) peak increases with temperature, and other peaks gradually weaken until disappearing, which suggests a uniform shift of crystal facet induced by thermal stress (Figure S10) [35]. Therefore, we have successfully prepared the (1 0 0) fully preferred copper foil by low-temperature annealing of commercial copper foil.

The copper-supported tin interface layer was synthesized by immersing the commercially randomly oriented copper (R-Cu) and F-Cu foil in a tin salt solution for a short duration (Figure 2(e), Movie S1). As shown in Figure S11-S13, the chemical plated tin on R-Cu (R-Sn@Cu) exhibits a rough surface morphology with different grain shapes, and the surface of chemical plated tin on F-Cu (F-Sn@Cu) is relatively flat with uniform grain size and homogeneous thickness distribution (150 nm) after chemical plating for 2 min, which may be ascribed to uniform nucleation and growth on the uniformly oriented surface. The XRD patterns (Figure 2(f)) show that there is only a strong Bragg reflection of the tin (1 0 1) facet in the F-Sn@Cu foil, while the polycrystalline tin reflection and a weak copper-tin (CuSn) alloy peak is found in the R-Sn@Cu foil [36]. Since different crystal textures are realized at different reaction durations, the crystal orientation of the tin layer for a short-time chemical plating is further investigated by XRD and discussed in detail in Figure S14 and S15 [22]. The EBSD analysis also confirms the nearly single (1 0 1) facet in the F-Sn@Cu foil (Figure S16 and S17). The above characterization results prove that the copper-supported (1 0 1) fully preferred tin interface layer was successfully fabricated by a facile chemical tin plating method supplemented by low-temperature annealing. These approaches are potential to be extended for the industrial manufacture of unique current collectors (Figure S18).

The chemical tin plating on copper substrate is a replacement reaction process, in which the tin ions in the solution are deposited on the copper substrate, and the copper atoms in the bulk are dissolved into the solution (Figure 2(g)) [37–39]. The concentrations of tin and copper ions in plating solution are 3.46 g L^−1^ and 1.53 g L^−1^ after tin plating (Figure S19) (the concentration of tin ion is 5.26 g L^−1^ in the original tin plating solution), which indicates that tin ions in solution are partially reduced to tin metal layer on the copper substrate. First-principles calculation was applied to study the possible mechanism of oriented chemical tin plating (Figures 2(h) and 2(i) and Figure S20, S21). These differences in separation energy and binding energy lead to the kinetic heterogeneity in tin deposition on the different copper facets, which may be one of the important reasons for the formation of randomly oriented tin in R-Sn@Cu. Besides, the binding energy of tin on the copper (2 2 0) facet is the largest among all calculated facets, which may also facilitate the formation of CuSn alloy on the copper (2 2 0) facet during the chemical tin plating [40]. Furthermore, the surface energies of several tin facets were calculated to investigate the tin deposition morphology influenced by thermodynamics (Figure S22). The surface energy of the tin (1 0 1) facet is the lowest among the stable crystal surfaces in tin crystal. Therefore, the formation of tin layer with fully preferred orientation can be explained by the tendency of the uniform tin species adsorbed on the F-Cu foil to form a thermodynamically favorable surface with low surface energy during tin deposition owing to the uniform copper separation and tin adsorption on uniform-oriented copper during the chemical plating reaction [41, 42].

### 2.3. Electrochemical Performances of F-Sn@Cu

**Figure 3:**
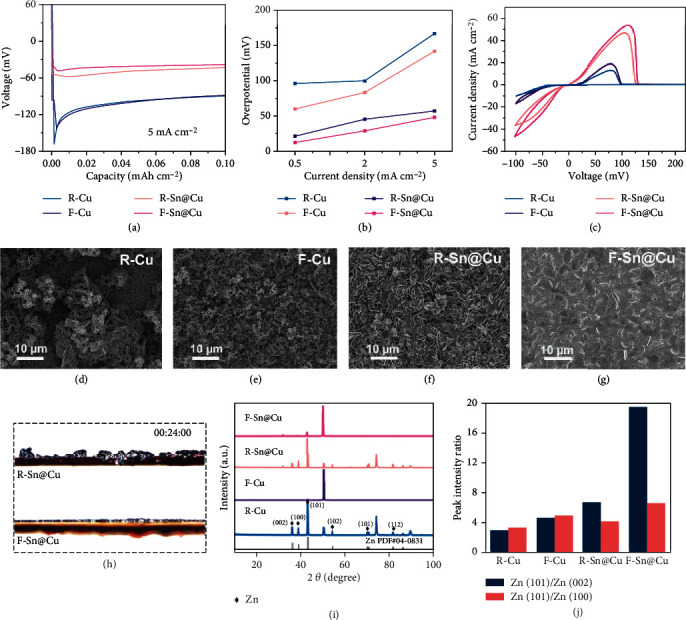
Zinc deposition behavior on F-Sn@Cu. (a) Voltage curves, (b) overpotential-current density curves, and (c) CV curves of R-Cu, F-Cu, R-Sn@Cu, and F-Sn@Cu. SEM images of (d) R-Cu, (e) F-Cu, (f) R-Sn@Cu, and (g) F-Sn@Cu after plating at 5 mA cm^−2^ for 2 mAh cm^−2^. (h) In situ optical observation of zinc deposition on R-Sn@Cu and F-Sn@Cu. (i) XRD patterns and (j) peak intensity ratios of (1 0 1)/(0 0 1) and (1 0 1)/(1 0 0) in zinc phase of the substrates after zinc plating.

The zinc deposition behavior was investigated in half cells with the as-prepared current collectors as working electrodes and zinc foils as the counter electrodes. As shown in Figure 3(a), there is a sharp voltage drop at the beginning of the zinc deposition, and subsequently a flat voltage plateau on the R-Cu and F-Cu surfaces. However, the voltage drop phenomenon is significantly diminished when the tin interface layer is introduced on the copper foil. Besides, the F-Sn@Cu substrate exhibits the lowest zinc plating overpotential over a wide range of current densities from 0.5 to 5 mA cm^−2^ (Figure 3(b) and Figure S23), which is ascribed to more high-activity areas participated in plating reaction. The overall interaction of zinc on the F-Sn@Cu surface is higher than that on the randomly oriented tin and copper substrates due to the strong binding of zinc with tin and the eliminated binding energy difference on the randomly oriented substrate, resulting in the low zinc deposition overpotential for the F-Sn@Cu [20, 22]. Zinc plating/stripping stability was further evaluated by cyclic voltammetry (CV) measurement (Figure 3(c)). The peak currents of substrates with fully preferred orientation (F-Sn@Cu and F-Cu) are higher than those of randomly oriented substrates (R-Sn@Cu and R-Cu) and their peak voltage gaps for zinc plating/stripping are narrower, which indicate a uniform and high-activity surface of F-Sn@Cu to reduce nucleation barriers and induce even zinc deposition [23, 43]. From the SEM images of as-prepared electrodes after zinc deposition (Figures 3(d)–3(g) and Figure S24-S26), zinc plating reaction can only occur at some specific active regions on the R-Cu, while the sparse deposits composed of zinc flakes cover the entire surface of F-Cu, which can be attributed to the selective deposition induced by the adsorption energy difference of zinc on the different facets of R-Cu foil. There is almost no deposition reaction on some facets of R-Cu surface due to the low zinc-binding energy of these facets. When crystal orientations of the substrate are uniform, entire zinc deposition can be observed on the surface with uniform zinc adsorption energy. The zinc deposition layer is relatively flat without inert region when introducing the tin interface layer onto the copper foil, but the R-Sn@Cu surface remains rugged with random orientations after zinc plating. In sharp contrast, the zinc plating surface of F-Sn@Cu is compact and no surface without deposition reaction can be observed. Besides, F-Sn@Cu shows the thinnest zinc plating layer among all the prepared substrates (Figure S25). The same result is also demonstrated by the real-time observation of the substrate electrode surfaces in a half cell with zinc anodes during the deposition process using in-situ optical microscope configuration (Figure S27 and Movies S2-5). To further clarify the role of substrate orientation in regulating zinc deposition, the binding energies of zinc on copper and tin surfaces were calculated by density functional theory (Figure S28). The (1 0 1)-oriented tin surface shows higher binding energy of zinc than other tin and the copper surface, which indicates strong interaction of F-Sn@Cu substrate with zinc, resulting in a more compact zinc plating layer. Besides, there are obvious binding energy differences of zinc on the different facets of randomly oriented tin and copper surfaces. In other words, the high zinc-affinity facet on the randomly oriented substrates can facilitate zinc deposition, while zinc deposition on the inert facet with low zinc affinity is far hysteretic. This phenomenon explains the rough surfaces on the substrate with random crystal orientation after zinc deposition. Zinc deposition on R-Cu with a half of tin-plated layer is investigated to further demonstrate the preference of zinc deposition on tin substrate over copper substrate. Optical photographs and SEM images (Figure S29) show that a large number of zinc are deposited on the tin-plated copper, but the bare copper exhibits a rough surface with few zinc protrusions, which indicates that tin is more favorable interface for zinc deposition. In the in situ optical observations for the sectional morphology (Figure 3(h), Figure S30, and Movies S6-S9), the local protrusions are formed in the early stage of zinc deposition and evolved into dendrites in the subsequent deposition on the surfaces of R-Cu and R-Sn@Cu, and then a large amount of dead zinc remains on the surface after zinc stripping, which further confirms the dendrite growth induced by the selective deposition on the randomly oriented surface. For the cells using F-Cu and F-Sn@Cu, zinc deposits exhibit a smooth surface with almost no dead zinc on the surface after complete zinc stripping. It can be seen from the XRD patterns of the oriented substrates after zinc deposition that the peak intensity ratios of both (1 0 1)/(0 0 1) and (1 0 1)/(1 0 0) increase with the transition of the substrate orientation from random to uniform (Figures 3(i) and 3(j)). The uniformly oriented interface can further promote the zinc deposition to grow toward the dominant (1 0 1) facet in comparison with the randomly oriented interface. This (1 0 1) facet-dominated zinc deposition can lead to a dense plating layer, as evidenced by the high peak intensity ratio in F-Sn@Cu. Nucleation mechanism is analyzed by a chronoamperometry (Figure S31), such as instantaneous nucleation and progressive nucleation. Both tin and copper metal substrates with and without uniform orientations follow an instantaneous nucleation mode, which suggests that zinc deposition quality on these substrates is greatly influenced by the initial nucleation. Besides, hydrogen evolution suppression and the electrical conductivity of tin substrate with fully preferred crystal orientation are greatly enhanced when compared with the random oriented tin and copper (Figure S32 and S33), which can contribute to the superior electrochemical performances for the F-Sn@Cu current collector. Therefore, it can be concluded that the crystal orientation and the metal affinity of the substrate both greatly affect zinc deposition. On randomly oriented substrates, zinc tends to be deposited on the highly active surface due to the difference in zinc adsorption energy on different facets, and the continuous deposition reaction inevitably leads to the dendrite formation. When crystal orientations of the substrate are uniform, the interaction between the zinc and substrate plays a dominant role in adjusting the quality of zinc deposition. Although zinc deposition can cover the entire surface of the F-Cu substrate, there is only a loose zinc plating layer with void space due to insufficient metal affinity. The high-quality zinc metal thin film anode obtained on the F-Sn@Cu substrate can be explained as a synergistic effect of the uniform surface crystal orientation and the high zinc affinity of the substrate. Besides, the enhanced electrical conductivity driven by uniform crystal orientation can also improve the zinc plating/stripping reversibility by facilitating electron transfer [44].

**Figure 4:**
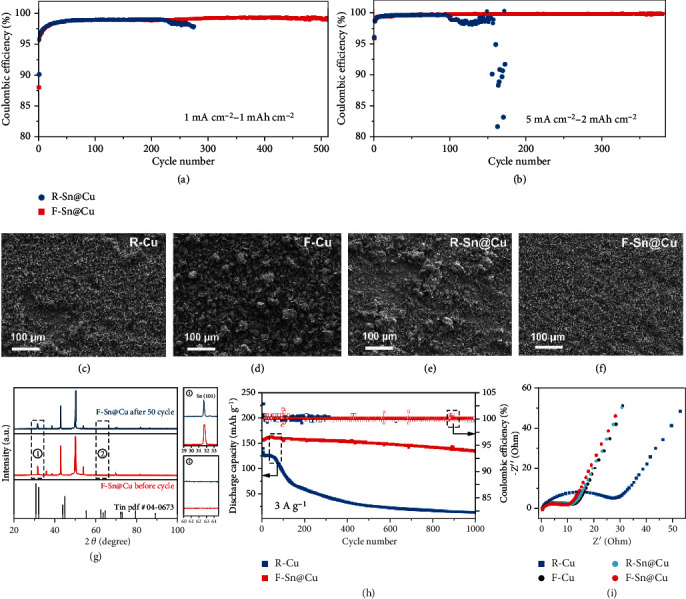
Electrochemical performance of F-Sn@Cu electrode in half-cells with a zinc counter electrode and full cells with NH_4_V_4_O_10_ cathode. CE of zinc plating/stripping on R-Sn@Cu and F-Sn@Cu at (a) 1 mA cm^−2^ for 1 mAh cm^−2^ and (b) 5 mA cm^−2^ for 2 mAh cm^−2^. SEM images of (c) R-Cu, (d) F-Cu, (e) R-Sn@Cu, and (f) F-Sn@Cu electrodes after 50 cycles. (g) XRD patterns of R-Cu, F-Cu, R-Sn@Cu, and F-Sn@Cu electrodes in the half-cell after 50 cycles. (h) Cycling performance of full cells using F-Sn@Cu and R-Cu anodes. (i) Nyquist plots of the full cells using R-Cu, F-Cu, R-Sn@Cu, and F-Sn@Cu anodes.

Cycling performance of the prepared current collectors was investigated in the half cell with zinc counter electrode. The cells using F-Sn@Cu current collector can deliver a stable Coulombic efficiency (CE) of 99.2% over 510 cycles at a current density of 1 mA cm^−2^ and capacity of 1 mAh cm^−2^, which is significantly improved in comparison to those of the R-Cu (98.6% after 30 cycles), F-Cu (99% after 60 cycles), and R-Sn@Cu (97.8% after 276 cycles) (Figure 4(a) and Figure S34). Even increasing the capacity to 2 mAh cm^−2^, F-Sn@Cu can be still operated stably for more than 380 cycles with the CE of 99.7% (Figure 4(b)). The excellent cycling performance of half-cell using F-Sn@Cu can be attributed to the stable and uniform surface of F-Sn@Cu, which is confirmed by their dendrite-free morphology with overall deposition after 50 cycles (Figures 4(c)–4(f) and Figure S35). Besides, the poststripped F-Sn@Cu after 50 cycles exhibits a flat surface without obvious dead zinc and by products (Figure S36). As shown in the XRD patterns of cycled substrates (Figure 4(g)), no significant difference is observed in F-Sn@Cu before and after cycling, illustrating that the repeated plating and stripping process cannot damage the crystal structure of the fully oriented tin layer and the F-Sn@Cu surface still maintain uniform tin (1 0 1) facet for favorable zinc deposition. No peaks related to zinc hydroxide sulfate (ZHS) by-products are observed on the oriented substrates (S-Cu and S-Sn@Cu) after cycling in comparison to the strong ZHS reflection in polycrystalline copper (R-Cu) and tin (R-Sn@Cu) interfaces (Figure S37). The low CE is mainly due to the continuous loss of active zinc in the zinc anode, such as the formation of insulating ZHS and dead zinc induced by the corrosion reaction and uneven plating/stripping on the interface. The uniform zinc deposition and stripping, the suppressed corrosion, and hydrogen evolution are realized when introducing a uniformly oriented metallic tin layer with high zinc metal affinity at the interface between copper and electrolyte. As a result, the F-Sn@Cu substrate can deliver steady long cycle life with high CE and low-voltage hysteresis (Figure S38). Similar tests were conducted in the half cell with F-Sn@Cu as the working electrode and lithium metal as the counter electrode to investigate the effect of the oriented tin interface layer on the lithium deposition, as described in supporting information (Figure S39-S41). Surprisingly, the F-Sn@Cu electrode exhibits significantly enhanced CE and cyclic stability of lithium plating/stripping in the half cell, which demonstrates the feasibility of this uniform-oriented tin decorated copper substrate in high-performance lithium metal batteries.

### 2.4. Full Cell Evaluation

The F-Sn@Cu anode with the zinc plating capacity of 2 mAh cm^−2^ was used to couple with NH_4_V_4_O_10_ cathode for demonstrating its practical application in full cells using 2 M ZnSO_4_ electrolyte (Figure S42). The full cell using F-Sn@Cu anode can steadily operate for 1000 cycles at a current density of 3000 mA g^−1^ with the capacity retention of 85.3%, much superior to those of R-Cu (9.8%), F-Cu (19.6%), and R-Sn@Cu (17.1%) (Figure 4(h) and Figure S43, S44). It is evident from impedance spectroscopy analysis that the charge-transfer resistance of the full cell using the F-Sn@Cu anode is significantly improved compared to the anode with random crystal orientation, which is attributed to the strong interaction between zinc and the uniformly oriented substrate with improved conductivity (Figure 4(i) and Table S2). F-Sn@Cu anode also exhibits improved electrochemical performance in the full cell when matched with polyaniline (PANI) cathode (Figure S45, S46, and Table S3), suggesting the uniform-oriented tin-modified copper current collector has broad application prospects as a host material for metal electrodes.

## 3. Conclusions

In summary, we discovered that substrate crystal orientation was one of the key reasons for the formation of zinc dendrite by experimental observations and theoretical simulations. A crystal orientation strategy was proposed by constructing a uniformly oriented substrate surface to elude the interfacial inhomogeneous dendrite growth. A copper-supported (1 0 1) fully preferred tin substrate was fabricated by facile low-temperature copper annealing and chemical tin plating technologies, which could induce an even electric field on the entire surface to realize a smooth and compact zinc plating layer with the advantage of high zinc affinity of tin. This unique current collector exhibited significantly improved reversibility of zinc plating/stripping in half-cells using zinc counter electrode and full cells using NH_4_V_4_O_10_ or PANI cathodes. The proposed strategies of substrate orientation regulation and metal affinity design can afford a new idea to realize dendrite-free metal anode for practical applications.

## Data Availability

The data that support the findings of this study are available from the corresponding author upon request.

## Supplementary Materials

Supplementary Materials The details of the method for the preparation and characterization of the materials are listed in the supporting information document. Figures S1-S46 and Tables S1-S3 are shown in the support information document. Movies S1-MS9 are displayed in support information in MP4 format.
