# Supplementary material for: Designing Zinc Deposition Substrate with Fully Preferred Orientation to Elude the Interfacial Inhomogeneous Dendrite Growth
Source: Research (Wash D C). 2022 Aug 18;2022:9841343. doi: 10.34133/2022/9841343 (PMC9422332; doi:10.34133/2022/9841343)
Supplement: Supplementary Materials — The details of the method for the preparation and characterization of the materials are listed in the supporting information document. Figures S1-S46 and Tables S1-S3 are shown in the support information document. Movies S1-MS9 are displayed in support information in MP4 format. [file 9841343.f1.zip › Xie_Supporting Information.docx]

Supporting information

**Designing zinc deposition substrate with fully preferred** **orientation to** **elude the interfacial inhomogeneous dendrite growth**

Chunlin Xie,^#^ Zefang Yang,^#^ Qi Zhang,* Huimin Ji, Yihu Li, Tingqing Wu, Wenbin Li, Pengfei Wu, Haiyan Wang*

*Hunan Provincial Key Laboratory of Chemical Power Sources, College of Chemistry and Chemical Engineering, Central South University, Changsha, 410083, P.R. China*

# These authors contributed equally to this work.

**Methods**

*Materials*

The specifical copper foil (30 µm, 99.99%) with a larger grain size was purchased from JingLiang Copper Industry. The commercially available copper foil (30 µm, 99.99%) and zinc foil (30 µm, 99.99%) were purchased from WanKeDa Metallic Materials. Ammonium persulfate ((NH_4_)_2_S_2_O_8_, AR) and aniline (C_6_H_7_N, AR), sodium sulfate (NaSO_4_∙H_2_O, AR), stannous chloride (SnCl_2_∙H_2_O, AR), thiourea (CS(NH_2_)_2_, AR), and sodium hypophosphite (NaH_2_PO_2_∙2H_2_O, AR) were purchased from Aladdin. Zinc sulfate (ZnSO_4_∙7H_2_O, AR), ammonium metavanadate (NH_4_VO_3_, AR), oxalic acid (H_2_C_2_O_4_·2H_2_O, AR), and hydrochloric acid (HCl, 36.5 *wt*%) were purchased from Sinopharm Group Co. Ltd. Electrolytes composed of Bis(trifluoromethane) sulfonamide lithium (LiTFSI), dimethoxyethane (DME), 1, 3-dioxolane (DOL) and LiNO_3_ for half cells with lithium counter electrode were purchased from Suzhou Duoduo Chemical Technology Co., Ltd.

*Fabrication of (2 0 0) fully preferred copper (F-Cu) foil*

The commercial copper foil was cut into 10×4 cm size and placed in the tubular furnace. The furnace was heated to 450 ºC for 4 h at a heating rate of 10 ºC min^−1^. The H_2_/Ar (H_2_ of 5 *vol*%) mixed atmosphere was maintained throughout the process.

*Fabrication of copper-supported (1 0 1) fully preferred tin (F-Sn@Cu) substrate*

F-Sn@Cu was fabricated by the chemical tin plating method according to the previous work.[1] The tin plating solution contains NaH_2_PO_2_∙2H_2_O (10 g L^−1^), CS(NH_2_)_2_ (70 g L^−1^), SnCl_2_∙H_2_O (10 g L^−1^), and hydrochloric acid (2 g L^−1^). First, the F-Cu foil was ultrasonically cleaned in dilute hydrochloric acid to remove the surficial oxide layer. The pretreated F-Cu was immersed into the above tin plating solution for different durations at room temperature and then washed with deionized water. The copper-supported randomly oriented tin (R-Sn@Cu) was prepared in the same procedure using commercially randomly oriented copper (R-Cu) foil as the tin plating substrate.

*Characterizations*

Scanning electron microscopy (SEM, JSM-7610FPlus) was used to observe the surface morphology of the tin-plated layer and zinc deposition. The scanning electron microscopes (Helios Nanolab G3 UC and Tescan Mira4 LMH) were used for the characterization of crystal orientation through electron back-scattered diffraction (EBSD). The argon ion polishing machine (1715610S) was used to pretreat copper foil for EBSD samples (5 kV, 200 µA, 10 min). An optical microscope (Nikon Ti2-A) with homemade mold was employed to in-situ observe zinc deposition. In-situ X-ray diffraction (XRD) was collected by a Bruker D8 X-ray diffractometer with a scanning rate of 4 º min^−1^. The ex-situ XRD data were tested by an Empyrean-2 diffractometer. Atomic Force Microscope (AFM, Bruker Dimension ICON) was used to observe the roughness of the electrode surface. Fourier transform infrared (FTIR) spectra were performed on a Thermo Scientific Nicolet iS5 spectrograph. The Inductively Coupled Plasma Optical Emission Spectrometer (ICP-OES, PerkinElmer 8300) was used to test the concentration of copper and tin species in the tin plating solution.

*Fabrication of NH_4_V_4_O_10_ and PANI cathode*

NH_4_V_4_O_10_ cathode was synthesized according to previous work.[2] In a typical synthesis process, NH_4_VO_3_ (1.1699 g) was first dissolved in deionized water at 80 ºC, then H_2_C_2_O_4_·2H_2_O (1.8912 g) was directly added to the above solution with continuous stirring until the solid was completely dissolved. After stirring for 30 min, the resultant solution was transferred into a Teflon-lined autoclave with stainless steel shell and reacted at 140 ºC for 48 h in an oven. After cooling, the black-green precipitate was collected by vacuum filtration, washed with deionized water, and dried in a vacuum drying oven at 80 ºC for 12 h. The polyaniline (PANI) was synthesized by an interfacial polymerization process.[3] (NH_4_)_2_S_2_O_8_ (0.183 g) and C_6_H_7_N (0.298 g) were dissolved in 10 ml HCl (1 M) solution, respectively. The above two solutions were mixed and kept stirring for 24 h. Then, the obtained precipitate was filtered and washed by deionized water and dried in a vacuum drying oven at 80 ºC for 12 h. The NH_4_V_4_O_10_ and PANI cathodes were fabricated by mixing the NH_4_V_4_O_10_ or PANI, acetylene black, and polyvinylidene fluoride with a weight ratio of 7:2:1 in NMP solvent. The resulting slurry was coated on stainless steel mesh and dried at 80 ºC for 12 h, and then cut into discs with a diameter of 11 mm. The active material loading was about 1.6 mg cm^−2^.

*Electrochemical measurements*

CR2025 coin-type cells were assembled for half cells and full cells with glass fiber as separator and 2 M ZnSO_4_ aqueous solution as the electrolyte. The galvanostatic charging-discharging test was carried out using the Neware battery test system at a constant temperature of 30 ºC. The electrochemical impedance spectroscopy (EIS) was performed on the Multi Autolab M204 electrochemical workstation with the frequency range of 0.01 Hz ⁓100 kHz. The cyclic voltammetry (CV) for half cells with zinc counter electrode was conducted on a CHI760E electrochemical workstation at a scanning rate of 1 mV s^−1^ with the voltage range of −0.1 ~ 1 V. The linear sweep voltammetry (LSV) curve was obtained through the CHI760 electrochemical workstation with the prepared substrate as the working electrode, Saturated Calomel Electrode (SCE) as the reference electrode, Pt mesh as the counter electrode and the 1 M Na_2_SO_4_ as the electrolyte, respectively. The scanning rate of LSV was set to 5 mV s^−1^.

The half cells using lithium metal as counter electrode and prepared substrates as work electrodes were assembled in an Ar-filled glovebox with water and oxygen contents <0.1 ppm. The electrolyte was composed of 1M bis(trifluoromethane) sulfonamide lithium (LiTFSI) dissolved in dimethoxyethane (DME) and 1, 3-dioxolane (DOL) solvents (1:1, v/v) with 2 wt% LiNO_3_ as additive. The CV curves were collected on a CHI760E electrochemical workstation at a scanning rate of 1 mV s^−1^ with the voltage range of −0.1 ~ 1 V.

*Density functional theory calculations*

The first-principles calculations were performed with the Vienna ab initio simulation package (VASP) with the frozen-core projector-augmented wave (PAW) method. The Perdew–Burke–Ernzerhof (PBE) form with generalized gradient approximation (GGA) was adopted to describe exchange and correlation potentials. An energy cutoff was set to 450 eV for plane wave basis expansion. The geometry optimizations are conducted until the energy is converged to 1.0 × 10^−5^ eV and the force of each atom is less than 0.045 eV Å^−1^.

The surface structure was simulated using a ten-layer tin surface with three-layer releasable atoms both at the top and bottom. Each surface was separated by a 15 Å vacuum layer. Surface energy (*γ*) was calculated by the following equation:

*γ* = (*E*_slab_ – *n* × *E*_bulk_) / 2*A* (1)

*E*_slab_, *E*_bulk_, *n*, and *A* represent the total energy of the tin surface, the energy of a single tin atom in the crystal, the number of tin atoms in the crystal, and the surface area, respectively.

The binding energy (*E*_b_) and separation energy (*E*_s_) were calculated using a five-layer 3×3 supercell and releasable top three-layer atoms for relaxation according to the following equations:

*E*_b_ = *E*_total_ − *E*_sub_ – *E*_atom_ (2)

*E*_total_, *E*_sub_, and *E*_atom_ represent the total energy of the copper (tin) surface combined with tin (zinc) atom, the energy of copper (tin) surface, and the energy of tin (zinc) atom, respectively.

*E*_s_ = *E*_after_ + *E*_atom_ – *E*_before_ (3)

*E*_before_, *E*_after_, and *E*_atom_ represent the total energy of the initial copper surface, the energy of the surface after copper atom separation, and the energy of copper atom, respectively.

*Finite element analysis simulations*

The tertiary current distribution physical field with the two- (2D) and three-dimensional (3D) transient model in COMSOL Multiphysics 5.6 was applied to simulate electric field surface with different exchange current densities (ECDs). The simulation cell was a rectangular box with a dimension of 50 mm × 50 mm at the base and a height of 30 mm. The initial zinc ions concentration was set to 2 M and the ion diffusion coefficient was 1.0 × 10^−10^ m^2^ s^−1^. For the randomly oriented surface, 3D electrode surfaces and 2D interfaces were divided into five and three regions, respectively, and different ECDs were assigned to these regions. In the plating process, the current density was set to 1 mA cm^−2^.


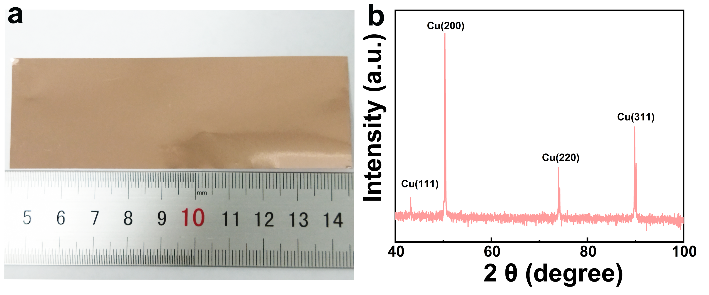


Figure S1 (a) Digital photograph and (b) X-ray diffraction pattern of specific commercial copper foil with large grain size.


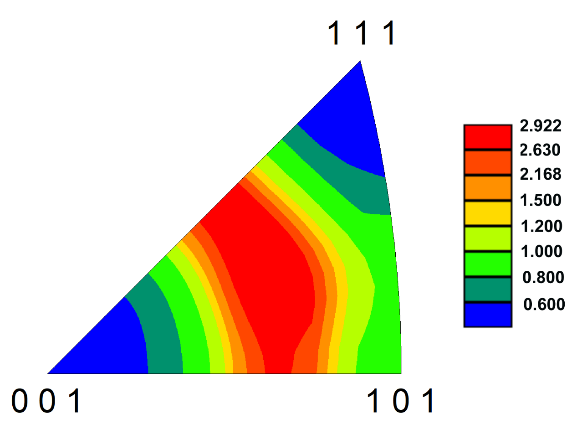


Figure S2 Inverse pole figure of [0 0 1] direction of the specific commercial copper foil with large grain size.

To investigate the effect of the copper surface with random orientation on zinc deposition, a specific commercial copper foil with a large grain size was applied as the deposition substrate because it is difficult to observe appearance changes affecting crystal orientation after zinc deposition on the common commercial copper foil with small grain size. The X-ray diffraction (XRD) pattern (Figure S1b) illustrates that the diffraction peaks of the specific copper foil are well assigned to (1 1 1), (2 0 0), (2 2 0), and (3 1 1) facets of the copper crystal.[4]


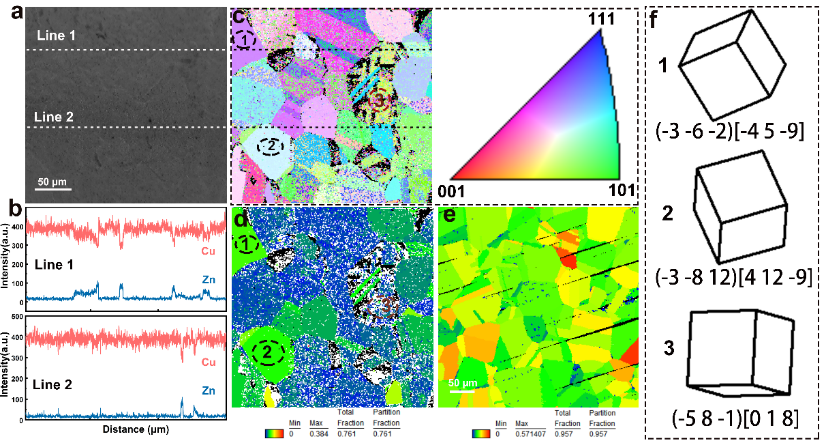


Figure S3 (a) SEM image, (b) the corresponding energy dispersive spectroscopy (EDS), (c) the inverse pole figure (IPF) map, and (d) the confidence index map of the specific commercial copper foil with large grain size after zinc deposition at 5 mA cm^−2^ for 20 s. (e) The confidence index map of the specific commercial copper foil before zinc deposition. (f) The crystal orientation of grains marked in Figure S3c.

Zinc deposition on randomly oriented copper foil was further investigated by SEM and EBSD. There is no obvious appearance difference on the different grains in SEM after zinc deposition due to the very low zinc deposition capacity. However, the significant zinc intensity differences between adjacent grains are observed by the linear energy dispersive spectroscopy, which suggests the uneven zinc deposition on the copper foil with random crystal orientation. It can be seen from the IPF map that some grains exhibit pure color (e.g. grains 1 and 2) while a large amount of noise points covers other ones (e.g. grain 3). Zinc deposition at the activated grain on the copper substrate can weaken the copper signal in EBSD, resulting in a large number of noise points on the copper grain covered by the deposited zinc, which indicates that the zinc deposition difference on different grains can be identified by the intensity of the copper EBSD signal in the confidence index map. Therefore, grain 3 with a weak EBSD signal facilitates zinc deposition while grains 1 and 2 with a relatively strong signal are not favorable to zinc deposition. Furthermore, the crystal orientations of the grains are indexed based on the Euler angle of the corresponding grain calculated by the OIM Analysis 7 software. The measured indices of the crystal orientation on grains 1, 2 and 3 are (-3 -6 2)[-4 5 -9], (-3 -8 12)[4 12 -9] and (-5 8 1)[0 1 8], which can be simplified to (1 1 2), (1 2 3) and (0 1 2) facets, respectively. Note that the extinction law in X-ray diffraction is not suitable for EBSD testing, that is, the extinction facet in X-ray diffraction can be displayed in EBSD.[5] It can be concluded that there exists a selective zinc deposition on the polycrystalline copper surface induced by crystal orientations, where the (1 1 2) and (1 2 3) facets of copper are not conducive to zinc deposition.

Table S1 The parameters of electric field simulations for zinc deposition on substrates with and without uniform orientation.

| Parameters | Symbol | Value | Units |
| --- | --- | --- | --- |
| Diffusion coefficient | *D* | 1.0 × 10^−10^ | m^2^ s^−1^ |
| Charge number | *z* | 2 | 1 |
| Initial concentration | *c* | 2.0 × 10^3^ | mol m^−3^ |
| Density | *ρ* | 7.14 | g cm^−3^ |
| Molar mass | *M* | 65.41 | g mol^−1^ |
| Applied current density | *I* | 1 | mA cm^−2^ |
| Exchange current densities | *I*_1_ | 5 | mA cm^−2^ |
|  | *I*_2_ | 10 | mA cm^−2^ |
|  | *I*_3_ | 8 | mA cm^−2^ |
|  | *I*_4_ | 5 | mA cm^−2^ |
|  | *I*_5_ | 2 | mA cm^−2^ |
|  | *I*_6_ | 16 | mA cm^−2^ |
|  | *I*_7_ | 17 | mA cm^−2^ |
|  | *I*_8_ | 18 | mA cm^−2^ |
|  | *I*_9_ | 19 | mA cm^−2^ |
|  | *I*_10_ | 20 | mA cm^−2^ |

To simulate zinc deposition on the randomly oriented surfaces with different ECD by the finite element method, different ECD are assigned to the different regions of the surfaces. The *I*_1_ is the ECD of uniformly oriented surfaces. For dendrite growth on the randomly oriented surfaces, *I*_2_ to *I*_5_ is the ECD of high-activity and low-activity regions for the plating side, respectively, and *I*_1_ corresponds to the ECD of the stripping side. Besides, to visualize the non-uniform electric field distributions on randomly oriented interfaces after electrochemical cycling, the ECD of *I*_6_ to *I*_10_ are assigned to different regions on the three-dimensional surfaces. All simulations are performed in ZnSO_4_ electrolyte with an ion diffusion coefficient of 1.0 × 10^−10^ m^2^ s^−1^ and electrolyte concentration of 2.0 × 10^3^ mol m^−3^.


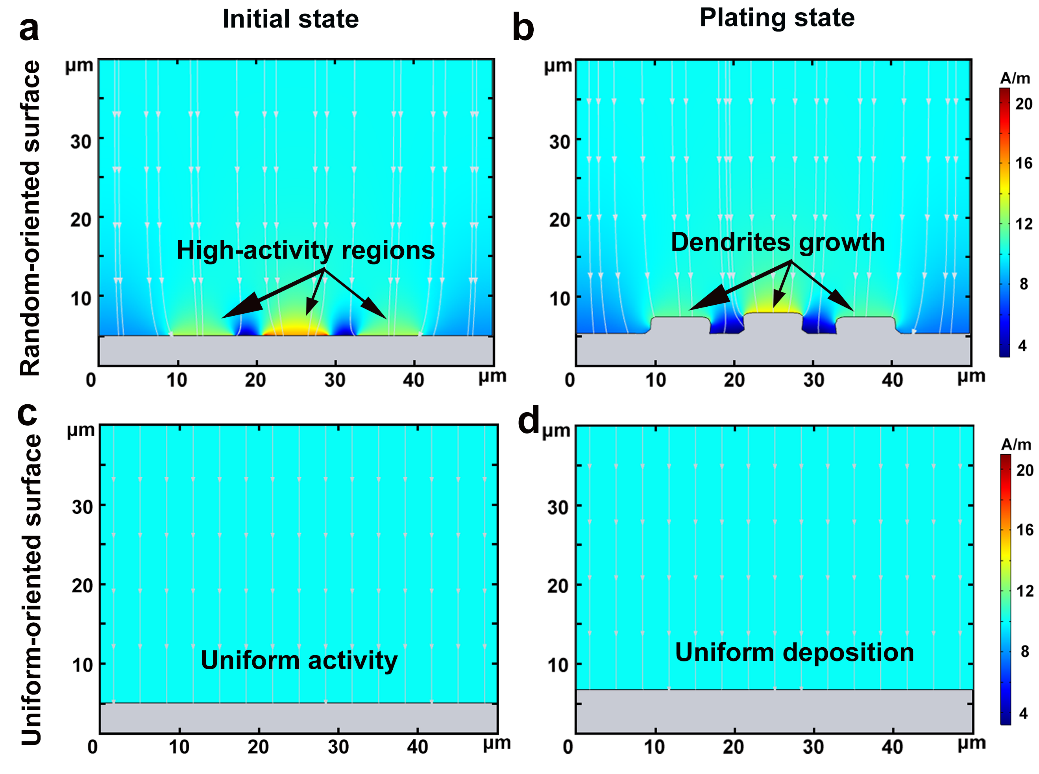


Figure S4 The electric field simulation of the surface with random and uniform orientation, (a, c) initial state, (b, d) plating state.





Figure S5 Voltage curve of the half cells using copper foil with random orientation as the cathode and zinc foil as the anode at 5 mA cm^−2^ for 2 mAh cm^−2^.

Copper-zinc half cells were assembled by the specific commercial copper foil with large grain size as the cathode and zinc foil as the anode to illustrate the cyclic stability of the randomly oriented copper foil. This copper-zinc half cell can operate for only 41 h.





Figure S6 XRD pattern of the common commercial copper foil after annealing at different temperatures.

The strongest peak of annealed copper transforms from (2 2 0) peak before annealing to (2 0 0) peak after annealing at 150 ºC. With temperature increasing, (1 1 1) peak disappears at 300 ºC and there is only (2 0 0) peak in the copper foil annealed at 450 ºC, which suggests that crystal orientations of common commercial copper foil are shifted toward (2 0 0) facet.


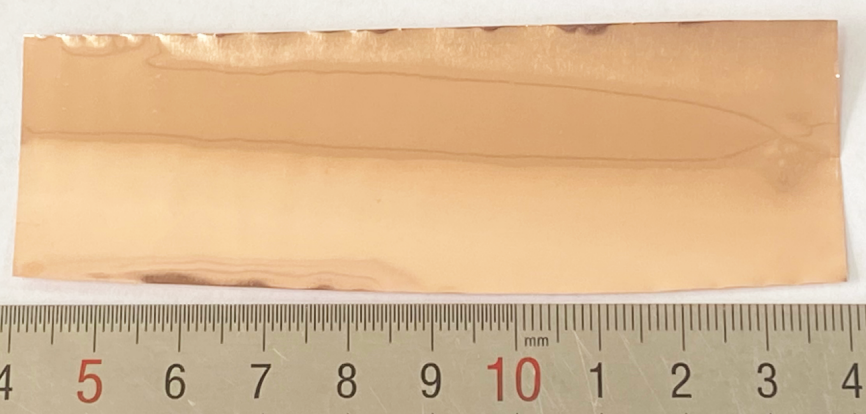


Figure S7 Digital photograph of the common commercial copper foil.


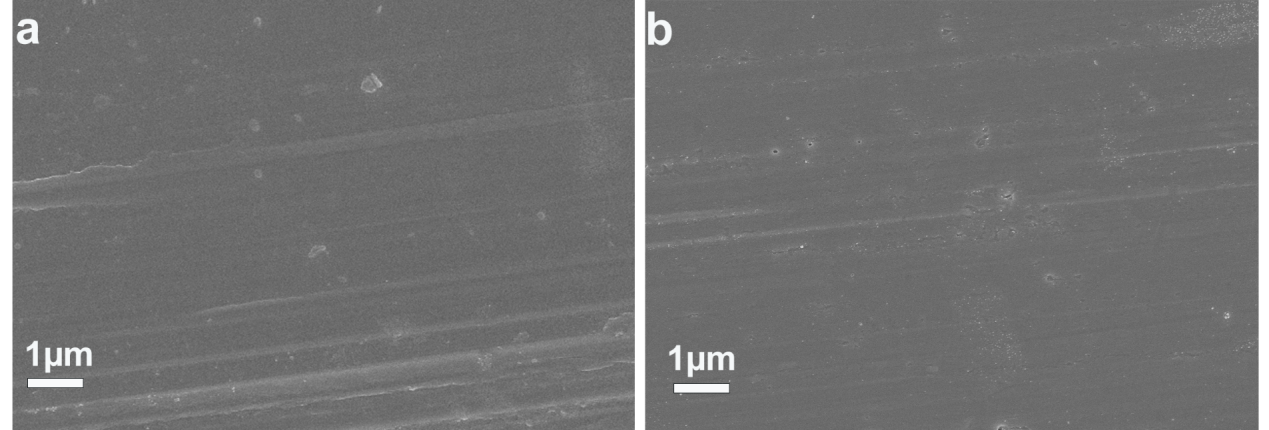


Figure S8 SEM images of the common commercial copper foil (a) before and (b) after annealing at 450 ºC.


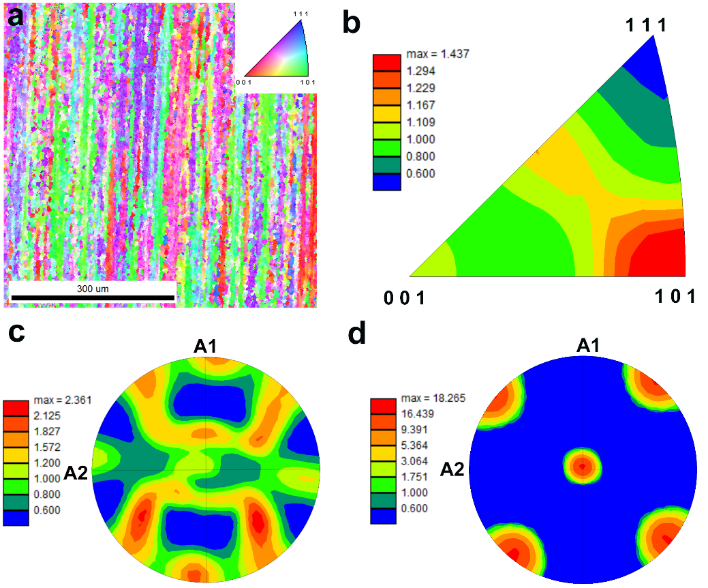


Figure S9 (a) IPF color map and (b) inverse pole figures in [0 0 1] direction of the common copper foil. (0 0 1) pole figures of the common copper foil (c) before and (d) after annealing at 450 ºC.


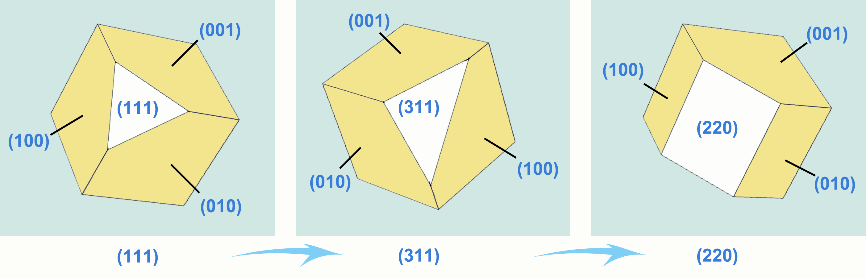


Figure S10 Crystal orientation shift processes of the copper foil driven by thermal stress. The white facets refer to the turning facets and the (1 0 0) facet is parallel to the copper foil surfaces.


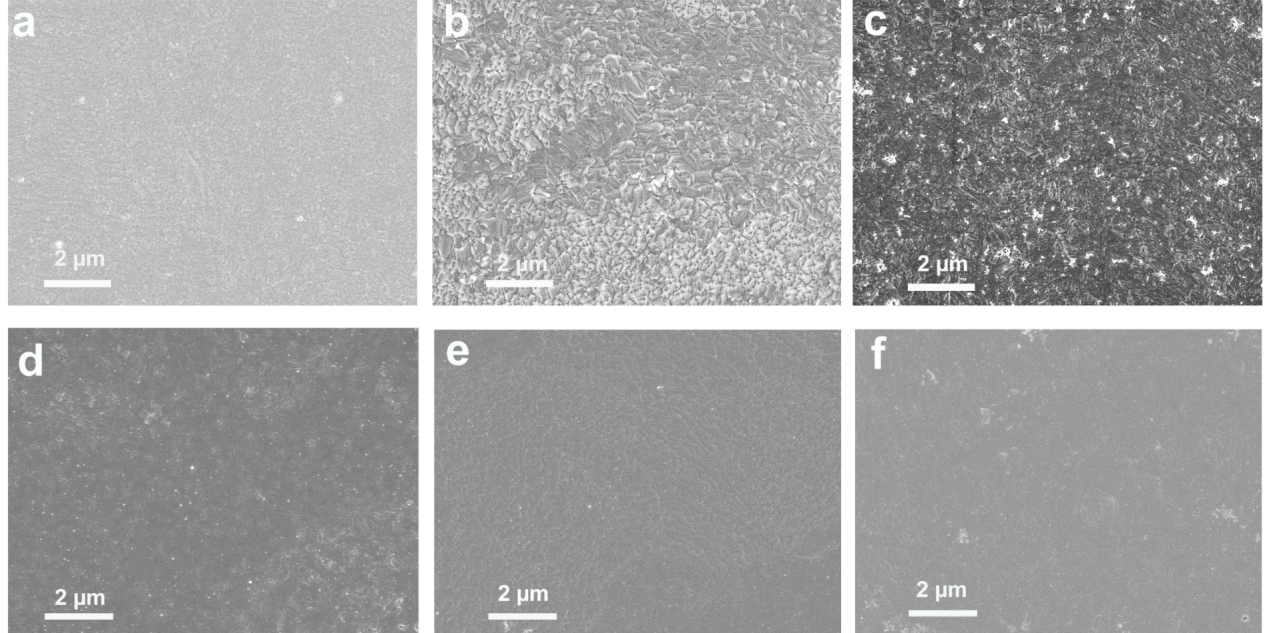


Figure S11 SEM image of chemical tin plating on (a-c) R-Cu and (d-f) F-Cu for 30 s, 2 min, and 4 min.

The surface morphology of R-Cu and F-Cu after chemical tin plating at different times is observed by SEM. There is no obvious difference in the tin deposition layer at 30 s between these two substrates. However, when the plating time increases to 2 min, the surface of R-Cu shows a greatly rough morphology with various grain shapes. In contrast, F-Cu after tin plating exhibits a smooth surface with uniform grain sizes. Even with increasing plating time, flat and dense surfaces can be still observed on the F-Cu in comparison with the rough R-Cu surfaces with void spaces.


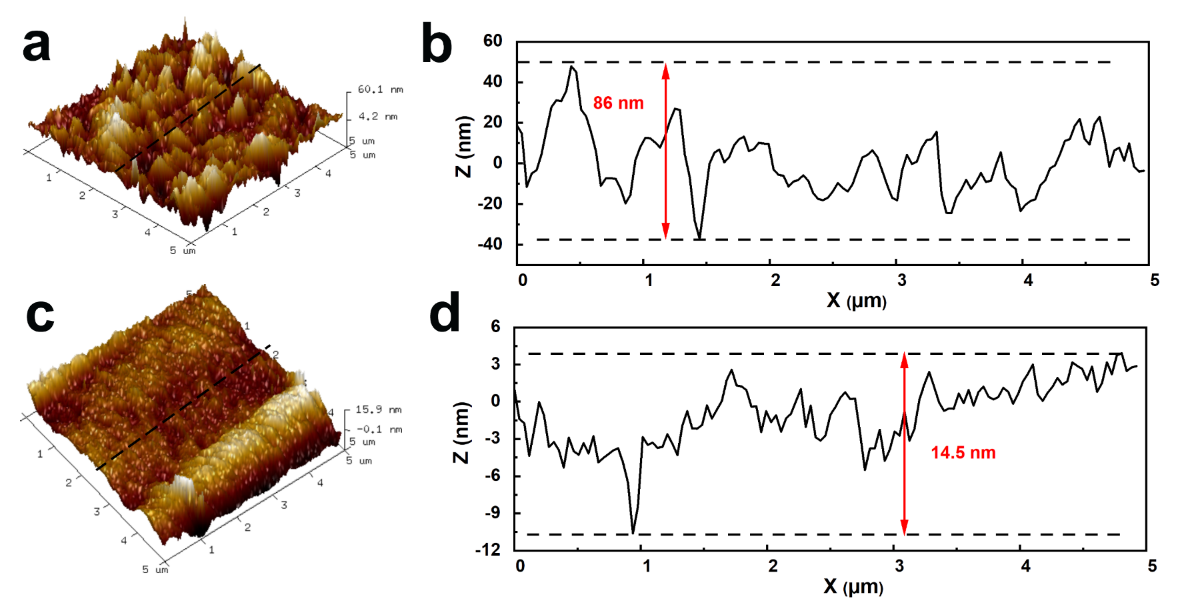


Figure S12 Atomic force microscope images and the corresponding height profiles of (a, b) R-Cu and (c, d) F-Cu after chemical tin plating for 2 min.


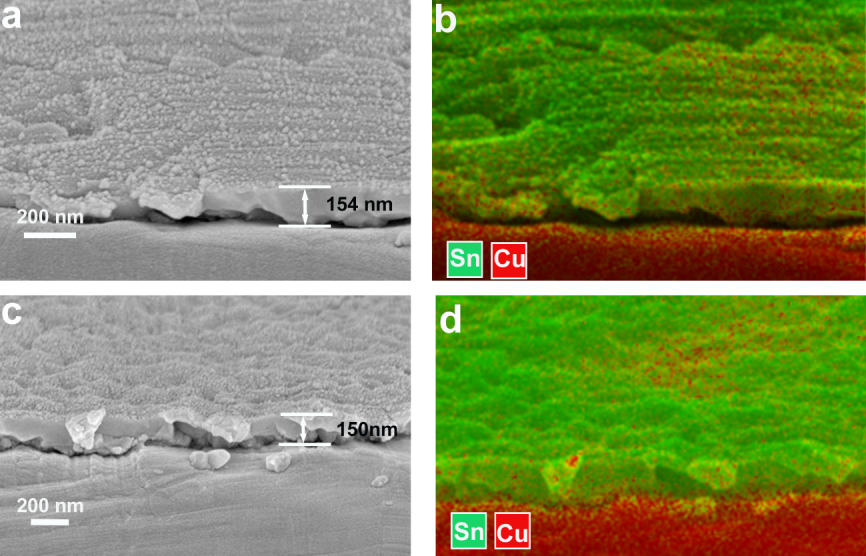


Figure S13 Sectional SEM images and corresponding EDS mapping of chemical tin plating on (a) R-Cu and (b) F-Cu foil for 2 min.





Figure S14 XRD pattern of R-Cu and F-Cu foil after chemical tin plating at 30 s.


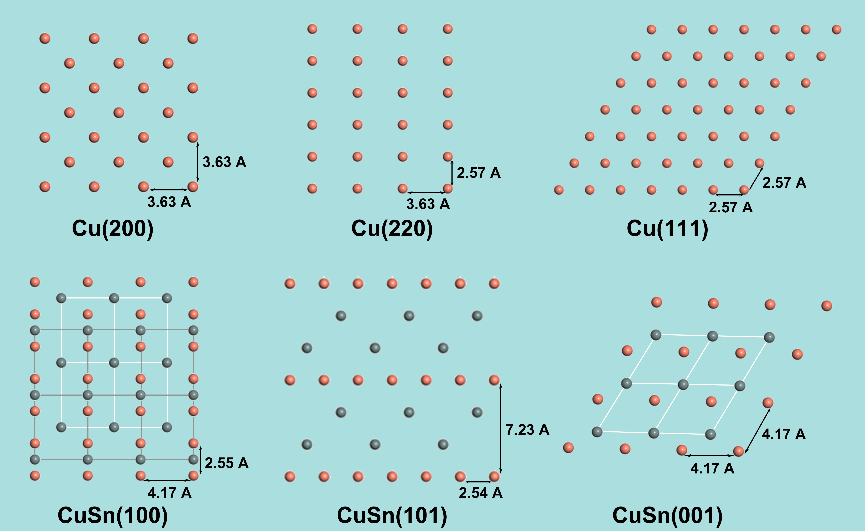


Figure S15 Structure comparison of different crystal facets in copper and copper-tin alloy.

The crystal structure of the tin layer after chemical plating for 30 s on the F-Cu foil was measured by XRD (Figure S14). It can be seen that the tin layer on the F-Cu foil after a short-time chemical plating process still shows only a single diffraction peak corresponding to the (1 0 0) orientation of copper, which demonstrates that the structure of tin plating on the uniform substrate cannot be changed with increasing reaction time. Besides, there always exists a CuSn alloy peak in the randomly oriented copper substrate after chemical tin plating, which may be attributed to the different orientations of the substrate surface.[6] The lattice parameters of different facets in copper and copper-tin alloy are compared in Figure S15. The results show that (1 0 0) facet of CuSn has a low lattice mismatch with copper (2 2 0) facet, suggesting that the chemical tin plating reaction on the copper (2 2 0) surface may facilitate the formation of CuSn alloy. It can be concluded that copper surface with (2 0 0) orientation is not a favorable facet for CuSn alloy formed by chemical tin plating.


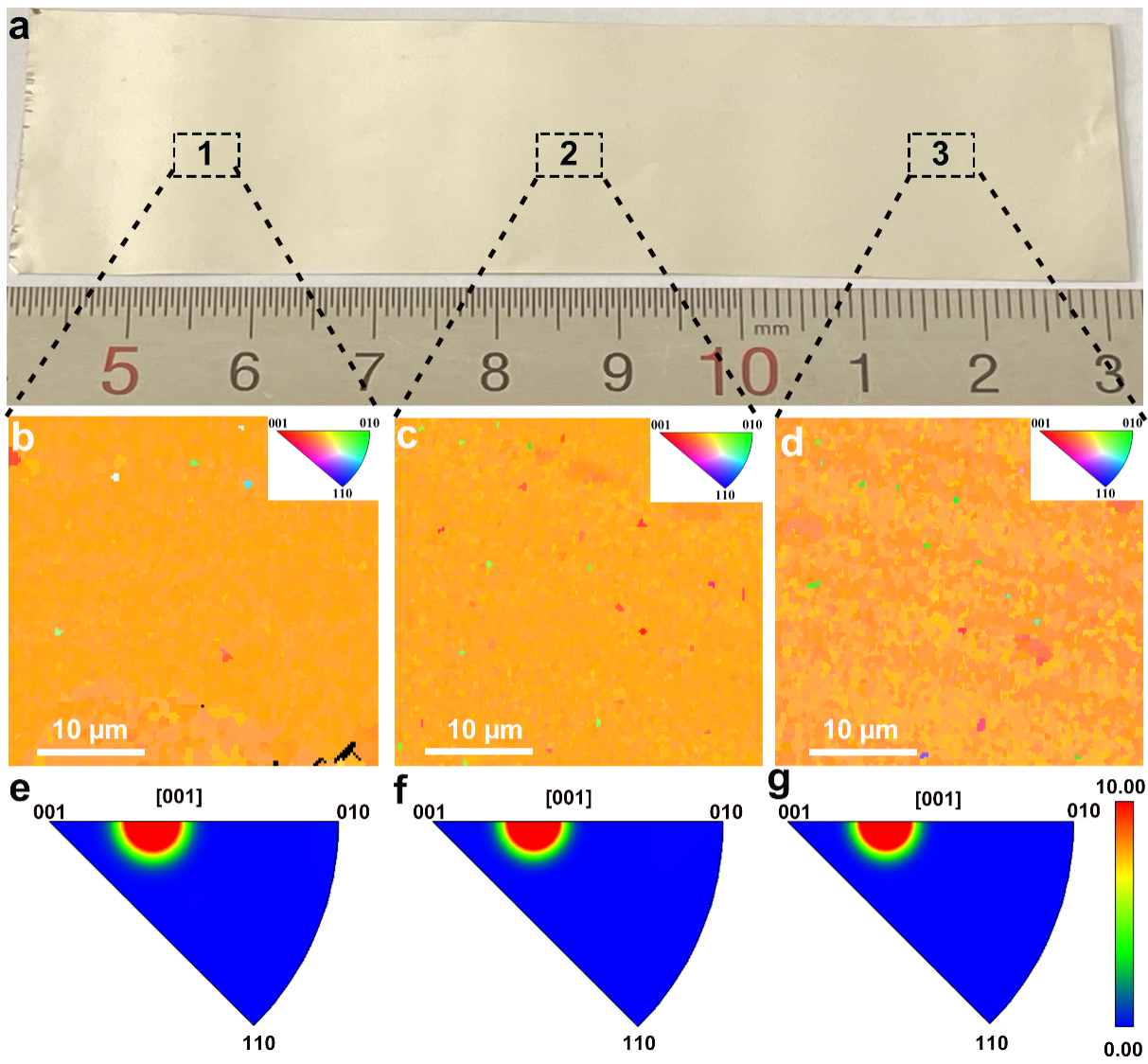


Figure S16 (a) Digital photograph, (b-d) corresponding IPF color map, and (e-g) the inverse pole figure of [0 0 1] orientations of F-Cu after chemical tin plating for 2 min.

The uniform color near orange in the IPF map of F-Cu after chemical tin plating indicates the same crystal orientation on the plated surface. The crystal orientation of the chemical tin-plated F-Cu foil is assigned to the tin (1 0 1) facet according to the reverse pole figure.


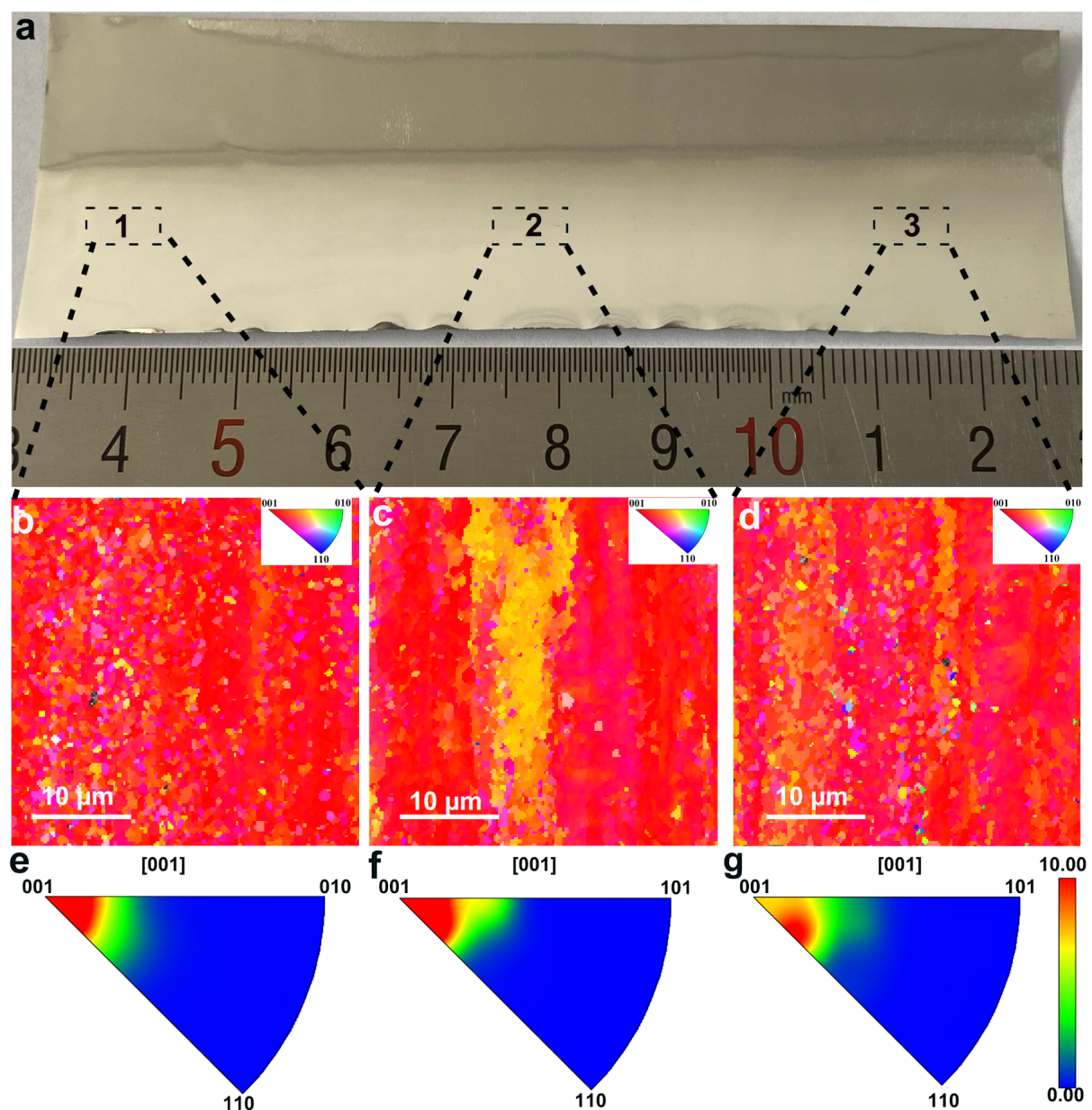


Figure S17 (a) Digital photograph, (b-d) corresponding IPF color map, and (e-g) the inverse pole figure of [0 0 1] direction of R-Cu after chemical tin plating for 2 min.

The R-Cu surface after chemical tin plating shows various colors in the IPF map, which indicates the random orientation on this polycrystalline surface.


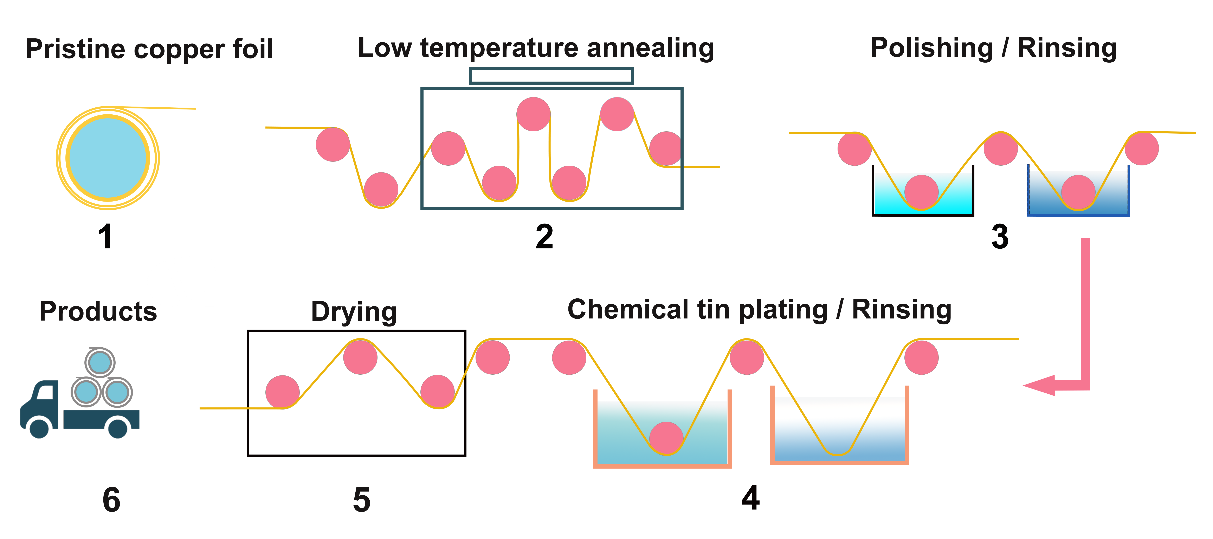


Figure S18 Schematic illustration of industrial manufacture of copper-supported (1 0 1) fully preferred tin current collector.


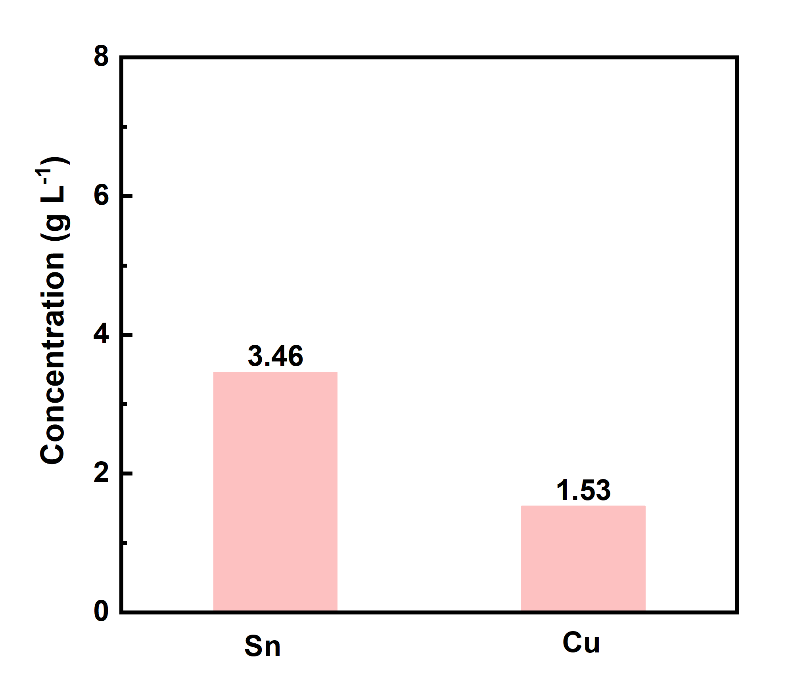


Figure S19 The concentrations of tin and copper ions in the plating solution after tin plating.


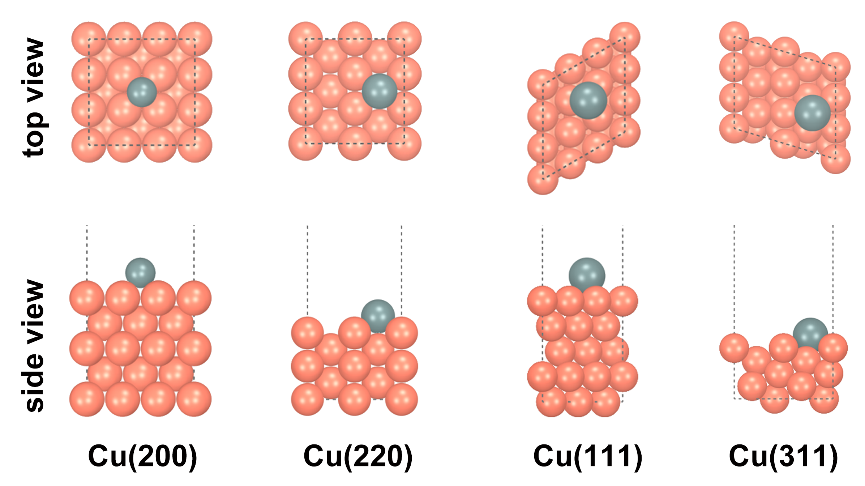


Figure S20 Calculation models of tin atoms adsorbed on different copper crystal facets, sphere filled in grey and orange refer to tin and copper atoms, respectively.


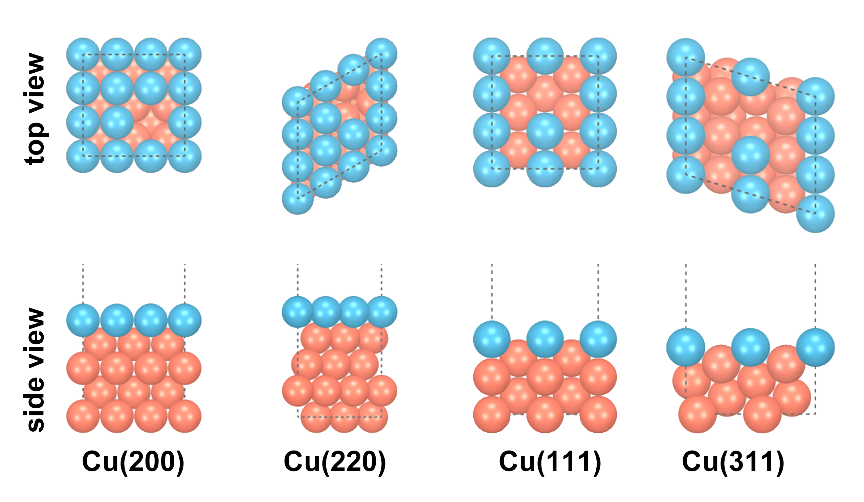


Figure S21 Calculation models of copper atoms separated from different copper crystal facets, sphere filled in light blue and orange refer to surficial and bulk copper atom, respectively.


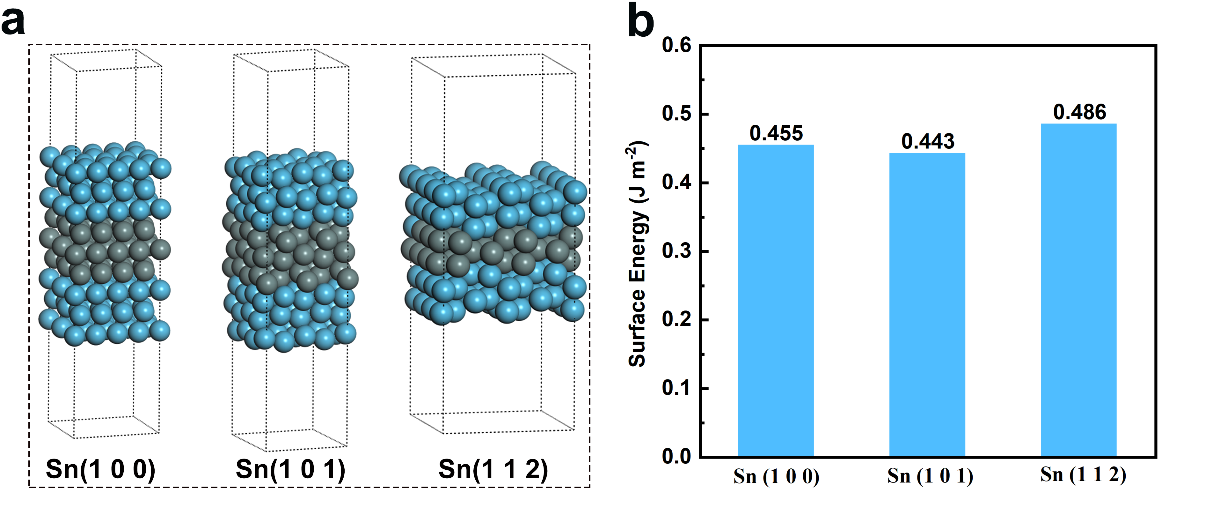


Figure S22 (a) Calculation models for tin surface energy and (b) the corresponding results, spheres filled in blue and grey refer to fixed and relaxed tin atom in the geometry optimization, respectively.





Figure S23 Voltage curves of R-Cu, F-Cu, R-Sn@Cu, and F-Sn@Cu in half cells with zinc anode at the current density of 0.5 and 2 mA cm^−2^.


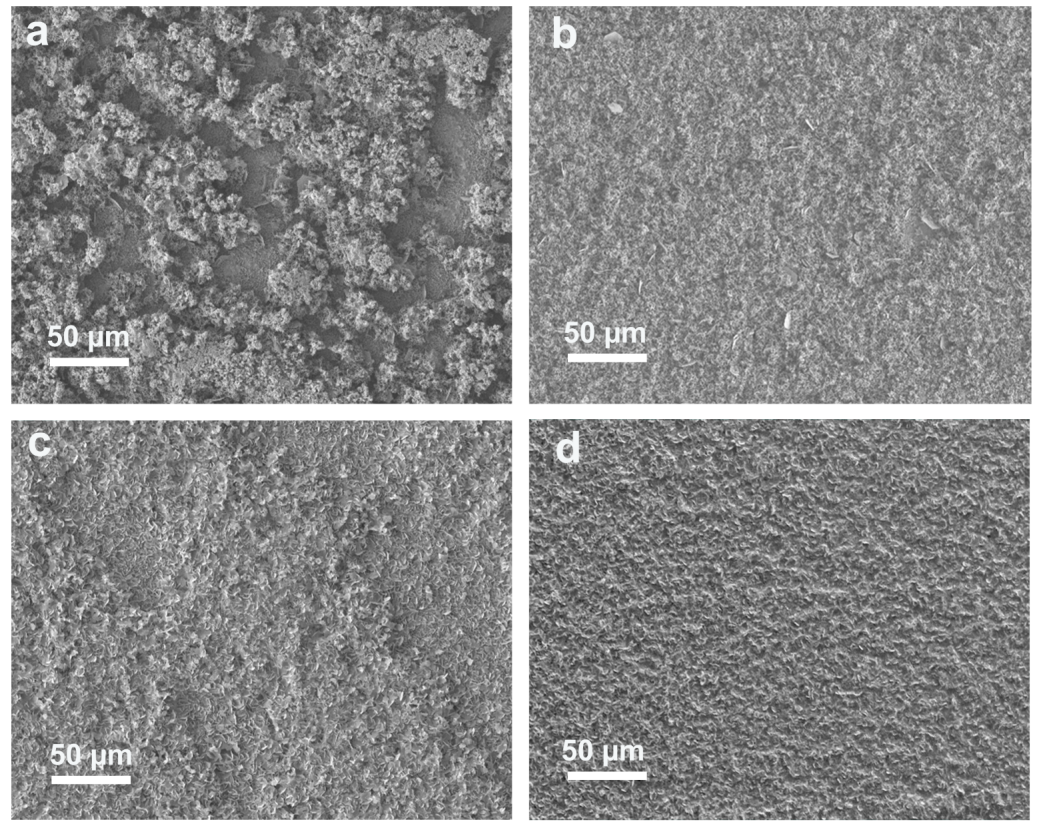


Figure S24 Surfacial SEM images of zinc deposition on the (a) R-Cu, (b) F-Cu, (c) R-Sn@Cu, and (d) F-Sn@Cu at 5 mA cm^−2^ for 2 mA cm^−2^.


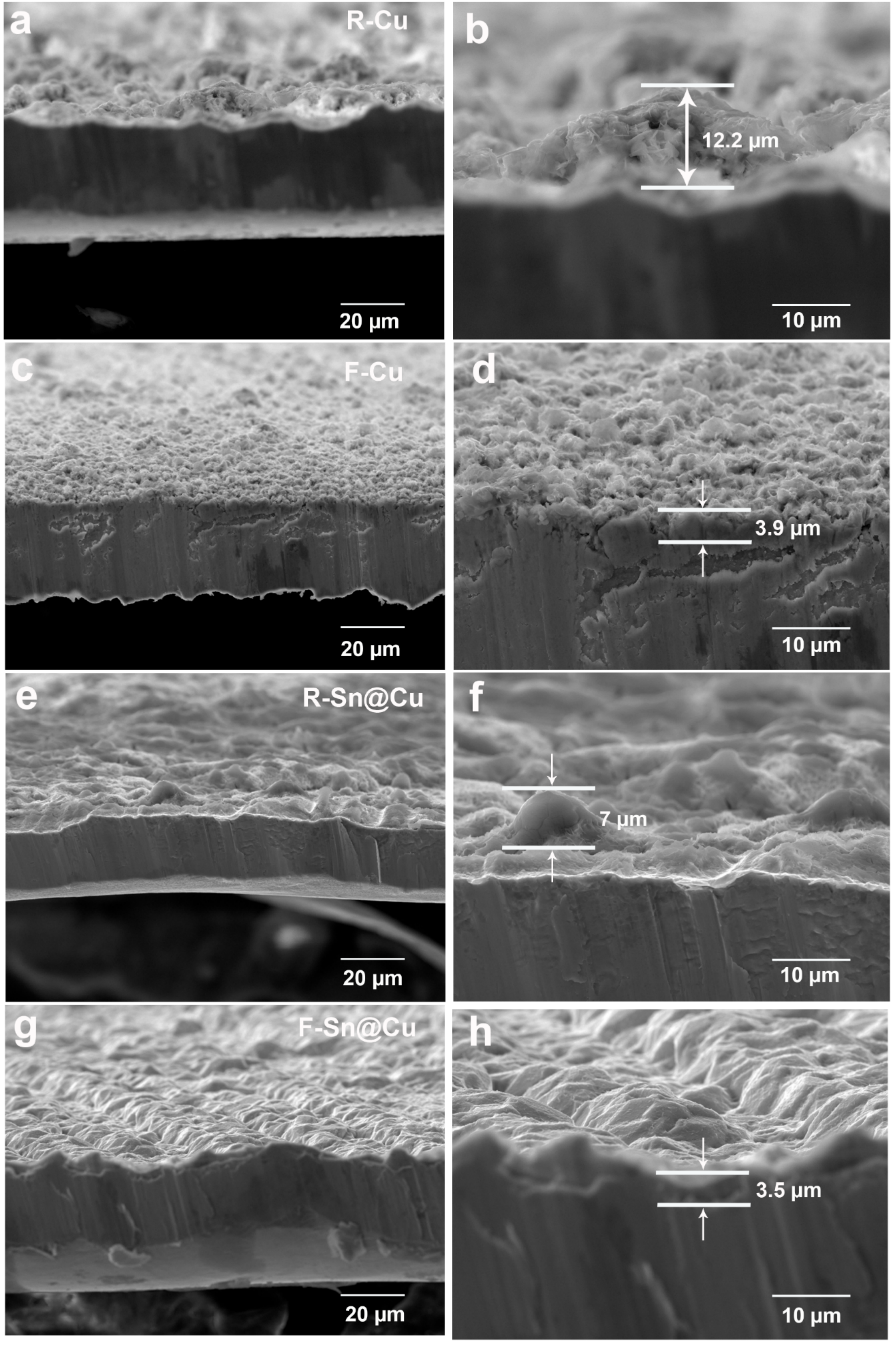


Figure S25 Cross-sectional SEM images of zinc deposition on the (a, b) R-Cu, (c, d) F-Cu, (e, f) R-Sn@Cu, and (g, h) F-Sn@Cu at 5 mA cm^−2^ for 2 mA cm^−2^.

Zinc deposition morphologies on the R-Cu, (b) F-Cu, (c) R-Sn@Cu, and (d) F-Sn@Cu are observed at a current density of 5 mA cm^−2^ and a maximum capacitor of 2 mA cm^−2^. R-Cu surface shows significantly uneven deposition and numerous inert regions without deposition reaction are observed. When the orientation of the R-Cu and R-Sn@Cu surfaces are uniformized, the overall deposition can be obtained on the F-Cu and F-Sn@Cu, indicating the eliminated facet-selective deposition on the randomly oriented surface. Besides, the thicknesses of zinc plating layer on oriented substrates (F-Cu and F-Sn@Cu) are lower than that on the polycrystalline substrates (R-Cu and R-Sn@Cu). There are no inert regions on the copper-supported tin interface after zinc deposition, which may be ascribed to the higher zinc affinity of tin in comparison to copper.[7] However, the surface of R-Sn@Cu after zinc plating is still rugged due to the different zinc deposition activity at the different facets on the surface with random orientation.


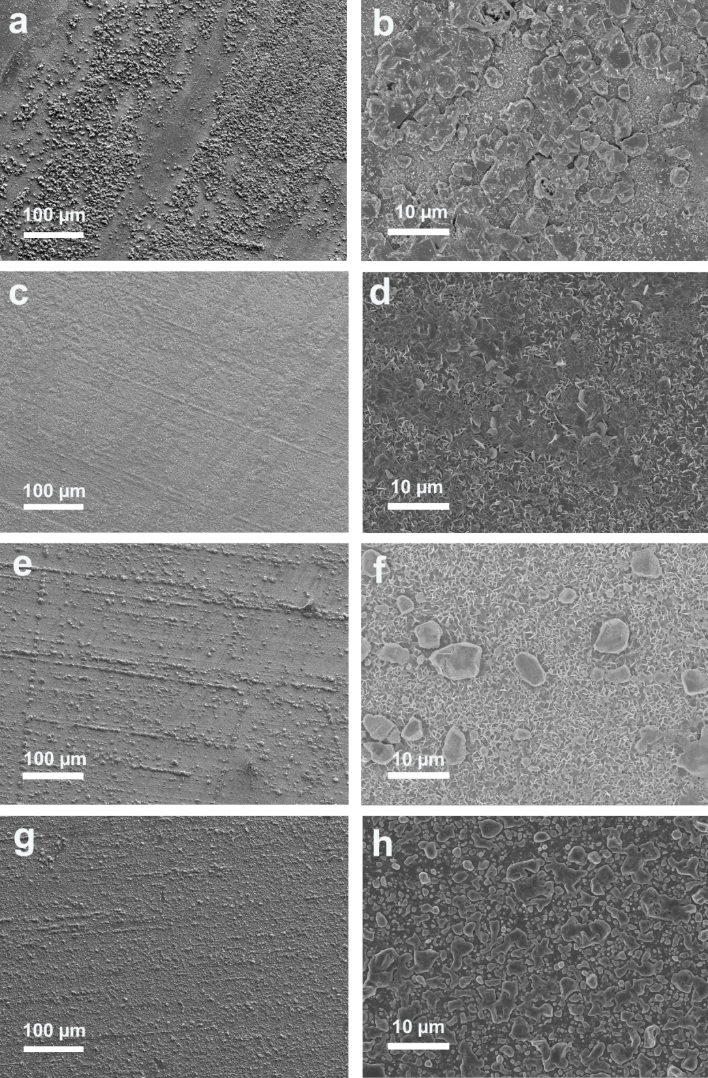


Figure S26 SEM images of zinc deposition on the (a, b) R-Cu, (c, d) F-Cu, (e, f) R-Sn@Cu, and (g, h) F-Sn@Cu at 5 mA cm^−2^ for 0.2 mAh cm^−2^.

To investigate zinc nucleation behavior on the oriented substrate at the plating infancy, the plating capacity is set to 0.2 mAh cm^−2^. As shown in Figure S23, uneven protuberance can be observed on the R-Cu and R-Sn@Cu surfaces, indicating that zinc ions are preferentially adsorbed at the high-activated facet on the randomly oriented surface to induce uneven nucleation. However, the F-Cu surface exhibits relatively even zinc nucleation with deposits composed of disordered zinc flakes covering the entire surface. A large amount of firmly rock-like zinc deposits are observed on the F-Sn@Cu surface profited from homogeneous zinc species adsorption induced by the uniformly oriented surfaces. These compact zinc deposits can induce homogeneous zinc growth in the subsequent deposition to obtain a thinner plating layer.[8] In the case of epitaxial growth, the formation of an even epilayer at the interface between different materials is a key factor in inducing homoepitaxial growth.[9] Therefore, the quality of zinc deposition may be influenced by the zinc deposits formed in the early stage.


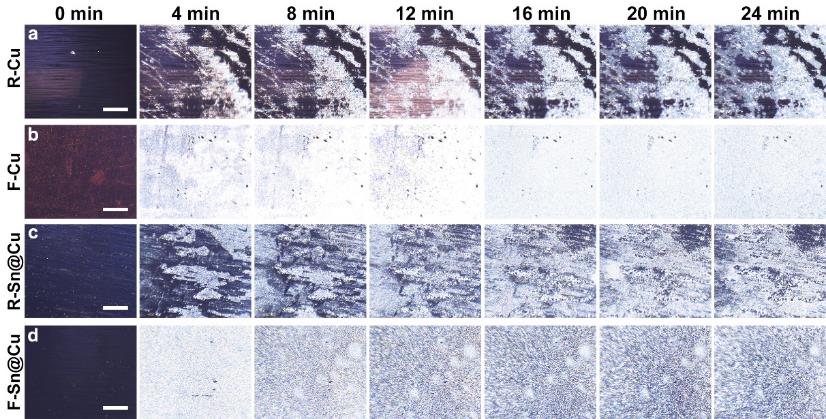


Figure S27 In-situ optical microscope surficial observations of zinc deposition on (a) R-Cu, (b) F-Cu, (c) R-Sn@Cu, and (d) F-Sn@Cu at 5 mA cm^−2^. Scale bars: 300 μm.


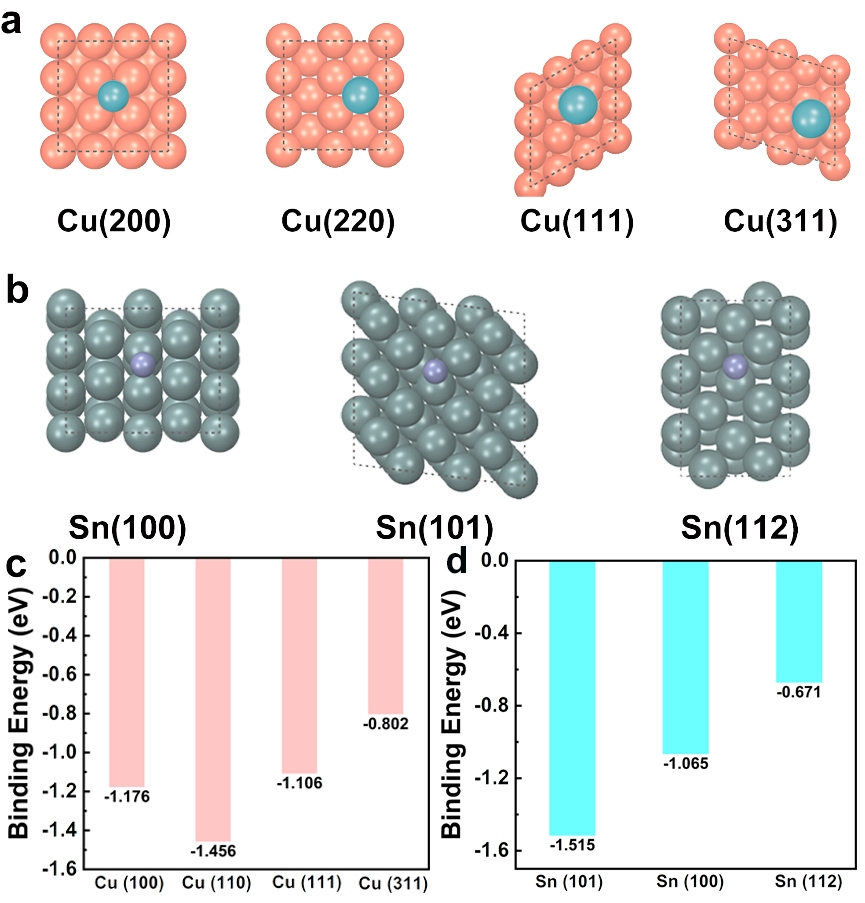


Figure S28 Calculation models (a, b) and the binding energy (c, d) of zinc on the different copper and tin surfaces, the sphere filled in light blue and violet refer to zinc atom, the sphere filled in orange and grey in bulk refer to copper and tin atoms, respectively.


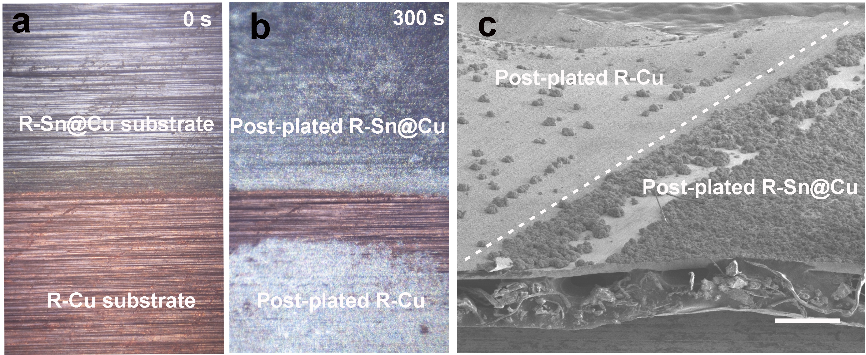


Figure S29 (a) Optical photograph of a piece of randomly oriented copper foil with half of the bare surface after chemical tin plating. (b) Optical photograph and (c) SEM images of R-Cu with half of the tin plating layer after zinc deposition at a current density of 5 mA cm^−2^ for 300 s.


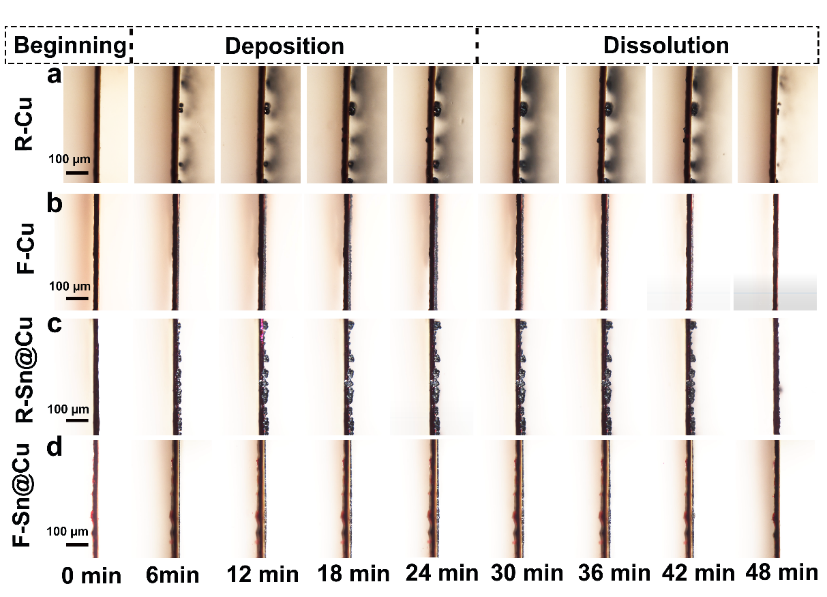


Figure S30 In situ optical microscope sectional observations of zinc deposition/dissolution processes on (a) R-Cu, (b) F-Cu, (c) R-Sn@Cu, and (d) F-Sn@Cu at 5 mA cm^−2^. Scale bars: 100 μm.


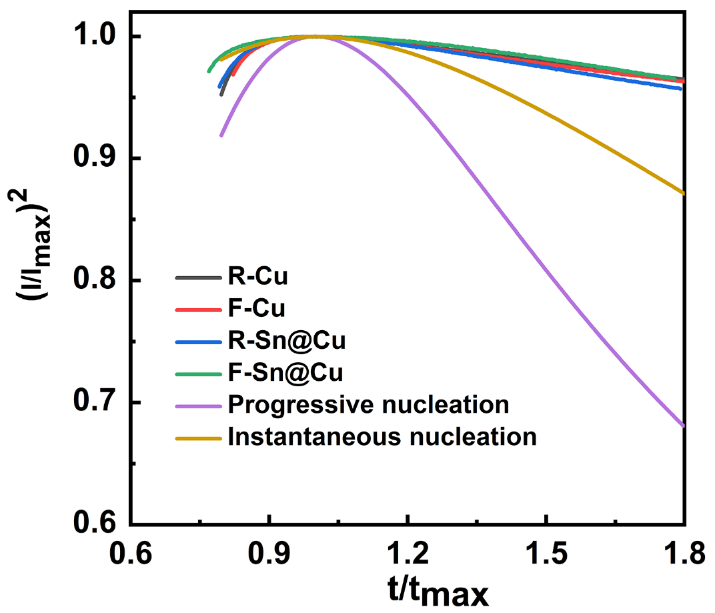


Figure S31 Dimensionless curves (I/I_max_)^2^ vs. (t/t_max_) for instantaneous and progressive nucleation derived from chronoamperometry.





Figure S32 The electrical conductivity of R-Cu, F-Cu, R-Sn@Cu, and F-Sn@Cu foil measured by the four-point probe.


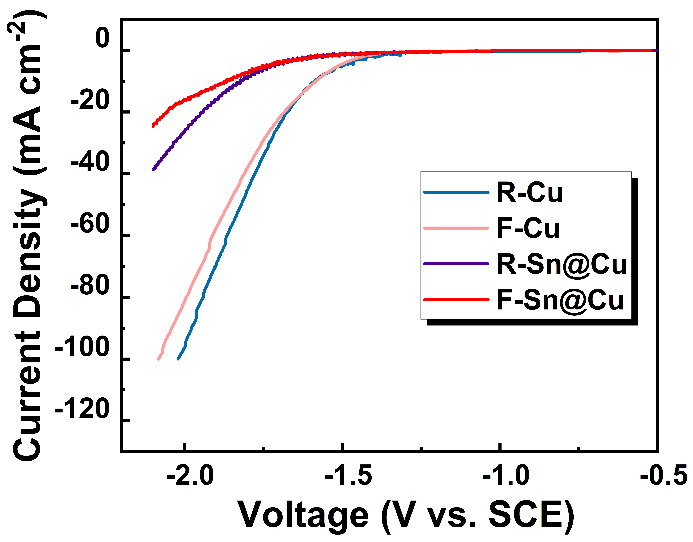


Figure S33 The linear sweep voltammetry curve of R-Cu, F-Cu, R-Sn@Cu, and F-Sn@Cu substrates in 1M Na_2_SO_4_ aqueous solution at 5 mV s^−1^.


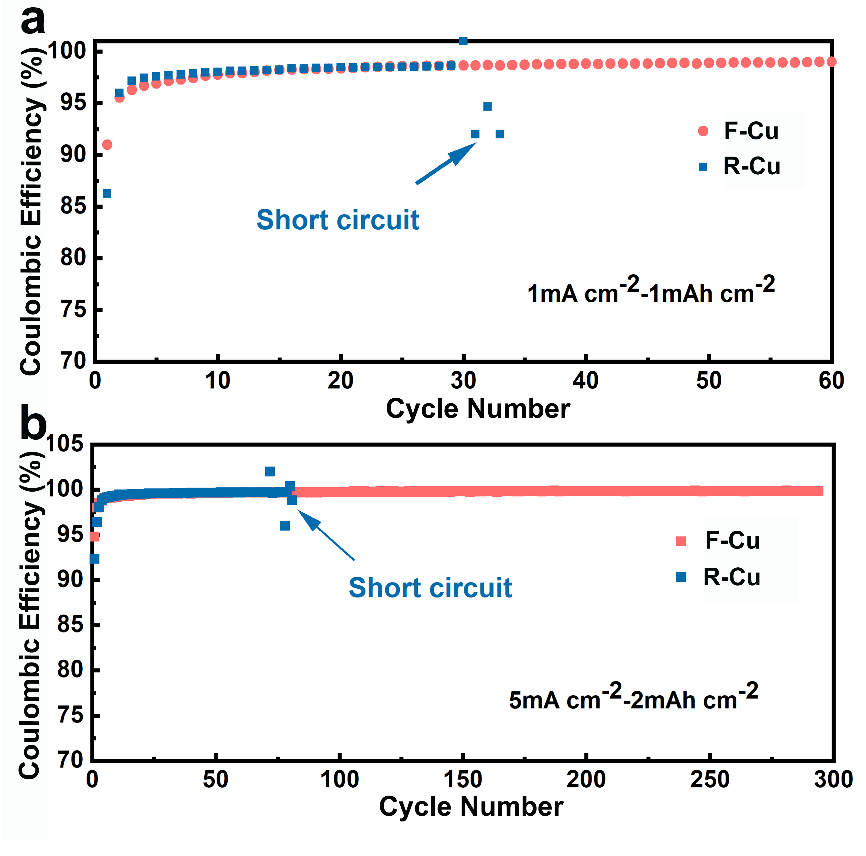


Figure S34 Cycling stability of R-Cu and F-Cu in a half cell with zinc counter electrode as the counter electrode.


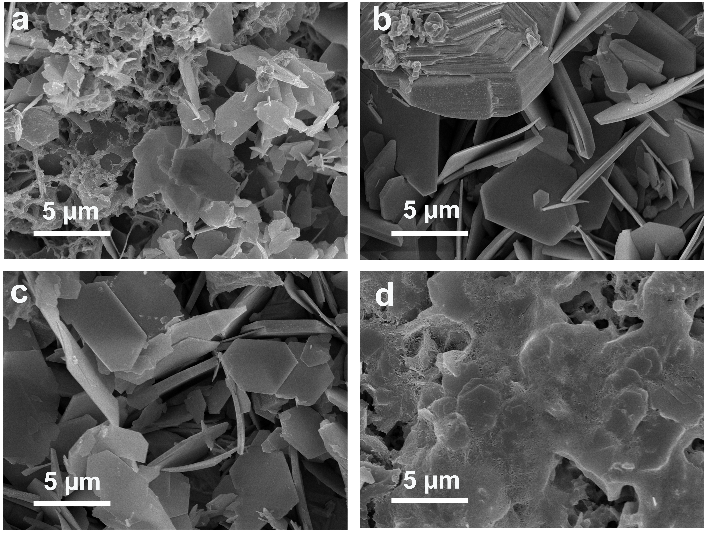


Figure S35 SEM images of the (a) R-Cu, (b) F-Cu, (c) R-Sn@Cu, and (d) F-Sn@Cu in a half cell with zinc counter electrode after 50 cycles at 5 mA cm^−2^ for 2 mAh cm^−2^.


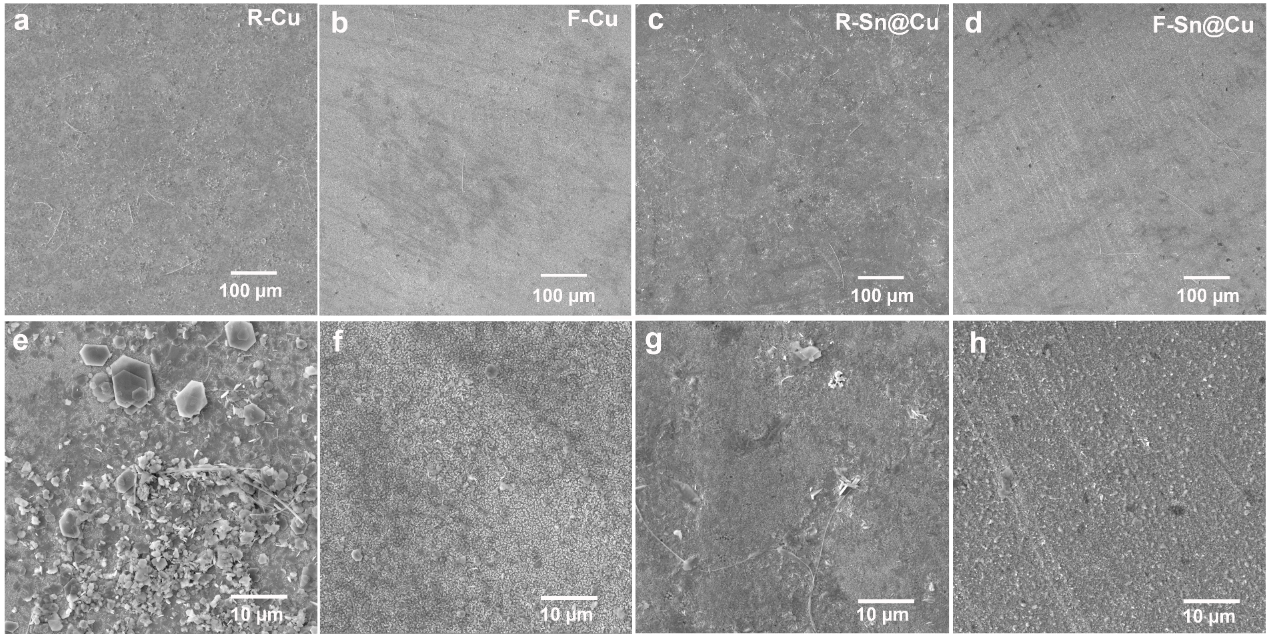


Figure S36 SEM images of the post-stripped (a) R-Cu, (b) F-Cu, (c) R-Sn@Cu, and (d) F-Sn@Cu in a half cell with zinc counter electrode after 50 cycles at 5 mA cm^−2^ for 2 mAh cm^−2^.


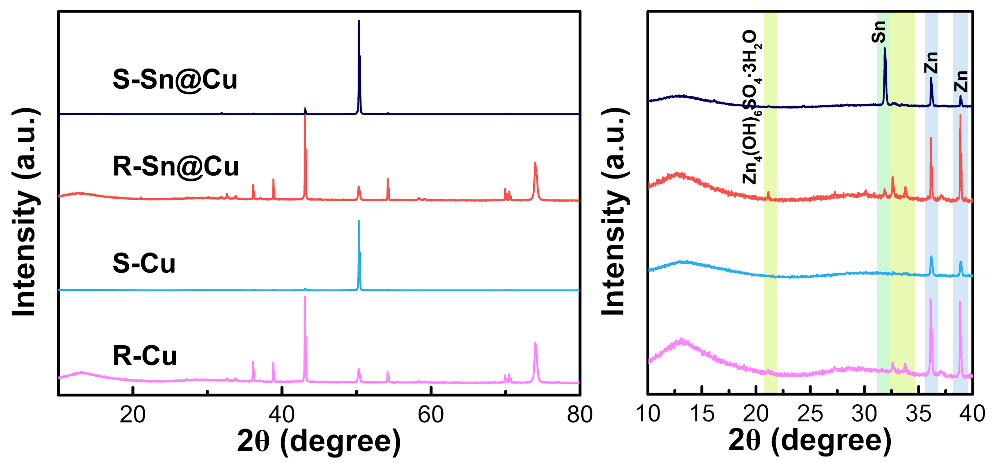


Figure S37 XRD patterns of R-Cu, F-Cu, R-Sn@Cu and F-Sn@Cu in a half cell with zinc counter electrode after 50 cycles at 5 mA cm^−2^ for 2 mAh cm^−2^.


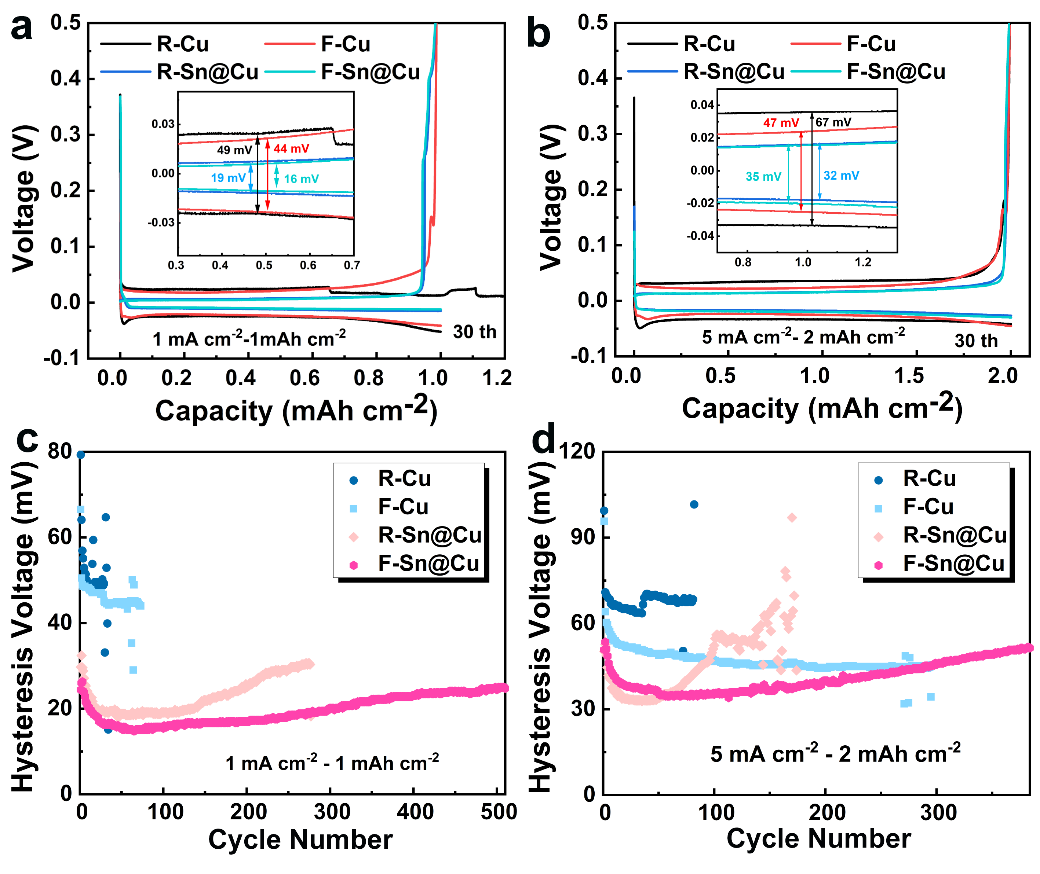


Figure S38 (a, b) Voltage curves at the 30th cycle and (c, d) voltage hysteresis of R-Cu, F-Cu, R-Sn@Cu, and F-Sn@Cu in half cells with zinc counter electrode.





Figure S39 CV curves of the half cells using R-Cu, F-Cu, R-Sn@Cu, and F-Sn@Cu as working electrode and lithium as counter electrode.

CV curves of R-Cu, F-Cu, R-Sn@Cu, and F-Sn@Cu in half cell with lithium counter electrode reveal the much higher activity and lower plating/stripping barrier of lithium deposition/dissolution on fully preferred substrates (F-Cu and F-Sn@Cu) than those of substrates with random orientation (R-Cu and R-Sn@Cu).[10, 11]





Figure S40 The CE of the half cells using R-Cu, F-Cu, R-Sn@Cu, and F-Sn@Cu as working electrode and lithium foil as a counter electrode at 1 mA cm^−2^ for 1 mAh cm^−2^.

Cycling performance of R-Cu, F-Cu, R-Sn@Cu, and F-Sn@Cu were evaluated in a half cell with lithium metal counter electrode at 1 mA cm^−2^ for 1 mAh cm^−2^. the cell using F-Sn@Cu can deliver CEs of 97.94% over 250 cycles, which is much enhanced compared to those of R-Cu (CEs of 74% upon 110 cycles), F-Cu (CEs of 98.7% upon 200 cycles), R-Sn@Cu (CEs of 86.2% upon 130 cycles). The significant improvement of the cycling performance in the F-Sn@Cu substrate is a result of the combined effects of the high lithium affinity provided by the (1 0 1)-oriented tin surface and uniform surface, which enables the overall nucleation and lithium growth.


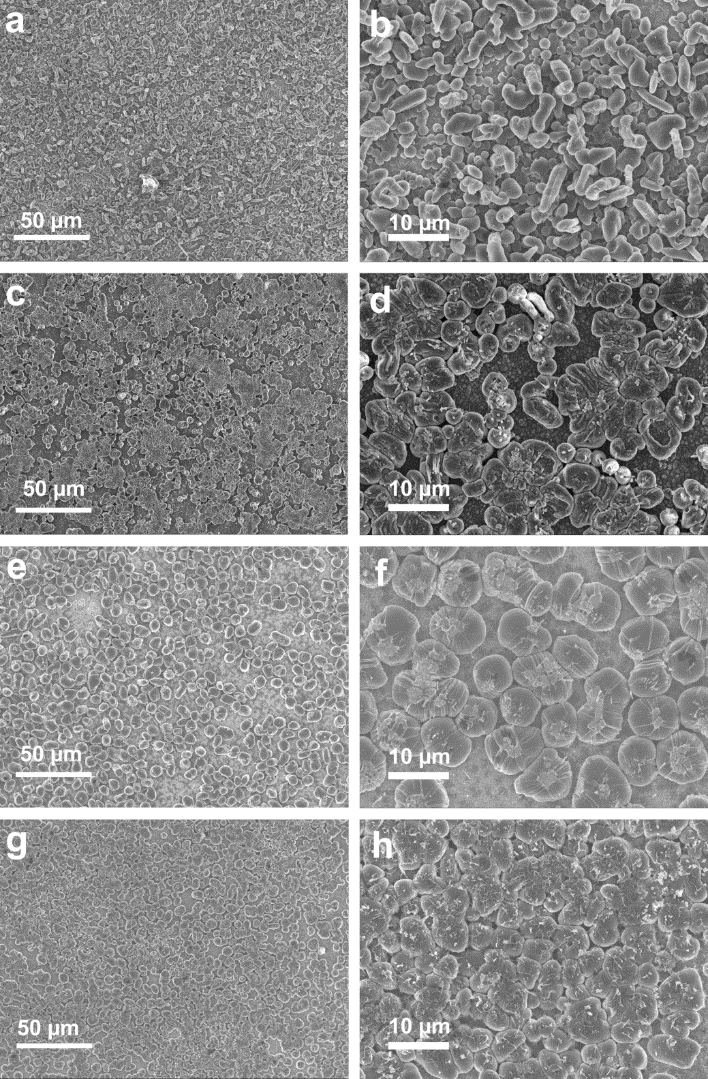


Figure S41 SEM images of the (a, b) R-Cu, (c, d) F-Cu, (e, f) R-Sn@Cu, and (g, h) F-Sn@Cu foil after lithium plating at 1 mA cm^−2^ for 1 mAh cm^−2^.

On the R-Cu surface, almost all lithium deposits are needle-like dendrites with a length of about 10 μm, while both spherical and needle-like lithium deposits can be observed on the F-Cu. When the tin interface layer is introduced onto copper foil, all lithium tends to be deposited with the spherical structure. Nevertheless, there are some regions without deposition reaction on the R-Sn@Cu after lithium plating. In contrast, lithium deposition on the F-Sn@Cu exhibits a smooth surface as the proportion of spherical lithium increases, which proves that a copper-supported fully preferred tin substrate can effectively improve metal lithium deposition.


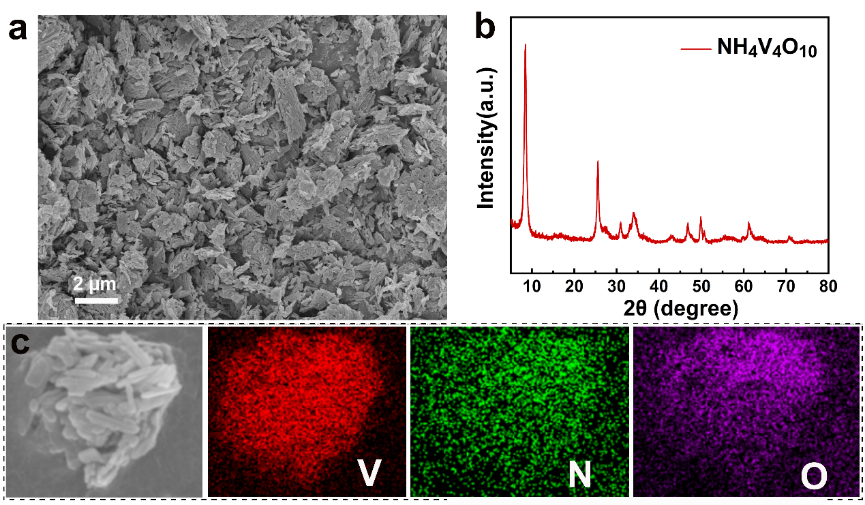


Figure S42 (a) SEM image, (b) XRD pattern, and (c) EDS mapping of NH_4_V_4_O_10_ cathode.





Figure S43 The discharge and charge curves for the 200th and 1000th cycles of full cells with NH_4_V_4_O_10_ cathode using (c) R-Cu, (d) F-Cu, (e) R-Sn@Cu, and (f) F-Sn@Cu plated with 2 mAh cm^−2^ of zinc as the anode.





Figure S44 Cycling performance of full cell with NH_4_V_4_O_10_ cathode using R-Sn@Cu and F-Cu plated with 2 mAh cm^−2^ of zinc as the anode.





Figure S45 (a) XRD pattern, (b) infrared spectroscopy, and (c, d) SEM images of the prepared PANI nanorod.





Figure S46 (a) CV curve, (b) the voltage profile, (c) the cycle performance, and (d, e) the impedance spectrum of the full cells with PANI as cathode and R-Cu, F-Cu, R-Sn@Cu, and F-Sn@Cu plated with 2 mAh cm^−2^ of zinc as the anode.

Table S2 The impedance parameters for full cells with NH_4_V_4_O_10_ cathode using R-Cu, F-Cu, R-Sn@Cu, and F-Sn@Cu plated with 2 mAh cm^−2^ of zinc as the anode.

| Samples | Rs | Rct |
| --- | --- | --- |
| R-Cu | 0.4 | 25.4 |
| F-Cu | 0.48 | 10.9 |
| R-Sn@Cu | 0.52 | 10.8 |
| F-Sn@Cu | 0.51 | 8.8 |

Table S3 The impedance parameters for full cells with PANI cathode using R-Cu, F-Cu, R-Sn@Cu, and F-Sn@Cu plated with 2 mAh cm^−2^ of zinc as the anode.

| Samples | Rs | Rct |
| --- | --- | --- |
| R-Cu | 0.59 | 149 |
| F-Cu | 0.52 | 20.8 |
| R-Sn@Cu | 1.43 | 7.1 |
| F-Sn@Cu | 0.84 | 3.9 |

References

[1] Q. Li, Y. Wang, F. Mo, et al., "Calendar life of zn batteries based on Zn anode with Zn powder/current collector structure," Advanced Energy Materials, vol. 11, no. 14, pp. 2003931, 2021.

[2] K.-F. Zhang, G.-Q. Zhang, X. Liu, et al., "Large scale hydrothermal synthesis and electrochemistry of ammonium vanadium bronze nanobelts," Journal of Power Sources, vol. 157, no. 1, pp. 528-32, 2006.

[3] J. Huang, and R. B. Kaner, "A general chemical route to polyaniline nanofibers," Journal of the American Chemical Society, vol. 126, no. 3, pp. 851-55, 2004.

[4] S. Jin, M. Huang, Y. Kwon, et al., "Colossal grain growth yields single-crystal metal foils by contact-free annealing," Science, vol. 362, no. 6418, pp. 1021-25, 2018.

[5] Z. Li, D. Yi, C. Tan, et al., "Investigation of the stress corrosion cracking behavior in annealed 5083 aluminum alloy sheets with different texture types," Journal of Alloys and Compounds, vol. 817, pp. 152690, 2020.

[6] A. Westgren, and G. Phragmén, "Röntgenanalyse der Kupfer–Zinnlegierungen," Zeitschrift für anorganische und allgemeine Chemie, vol. 175, no. 1, pp. 80-89, 1928.

[7] Z. Yang, C. Lv, W. Li, et al., "Revealing the two-dimensional surface diffusion mechanism for zinc dendrite formation on zinc anode," Small, pp. 2104148, 2021.

[8] Q. Zhang, J. Luan, Y. Tang, et al., "Interfacial design of dendrite-free zinc anodes for aqueous zinc-ion batteries," Angewandte Chemie International Edition, vol. 59, no. 32, pp. 13180-91, 2020.

[9] J. Zheng, Q. Zhao, T. Tang, et al., "Reversible epitaxial electrodeposition of metals in battery anodes," Science, vol. 366, no. 6465, pp. 645, 2019.

[10] L. Ma, Y. Ying, S. Chen, et al., "Electrocatalytic iodine reduction reaction enabled by aqueous zinc-iodine battery with improved power and energy densities," Angewandte Chemie International Edition, vol. 60, no. 7, pp. 3791-98, 2021.

[11] H. Park, A. Encinas, J. P. Scheifers, et al., "Boron-dependency of molybdenum boride electrocatalysts for the hydrogen evolution reaction," Angewandte Chemie International Edition, vol. 56, no. 20, pp. 5575-78, 2017.
